# Supplementary figures and images for: Oleanolic acid and moderate drinking increase the pancreatic GLP-1R expression of the β-cell mass deficiency induced hyperglycemia
Source: PeerJ. 2023 Jul 24;11:e15705. doi: 10.7717/peerj.15705 (PMC10373642; doi:10.7717/peerj.15705)

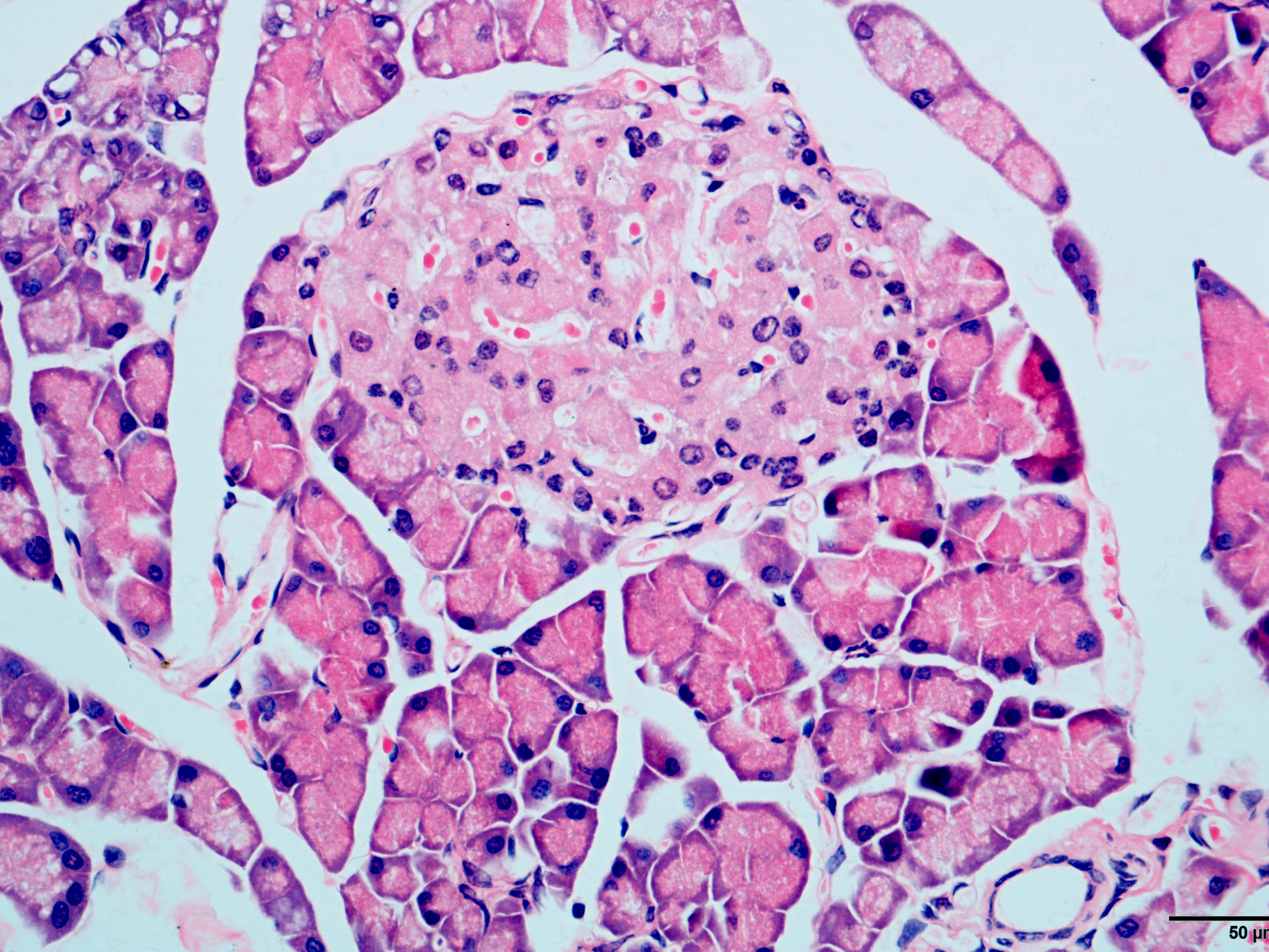

Supplement: Supplemental Information 2 [file peerj-11-15705-s002.zip › Fig.1 Physiology/Etoh.jpg]

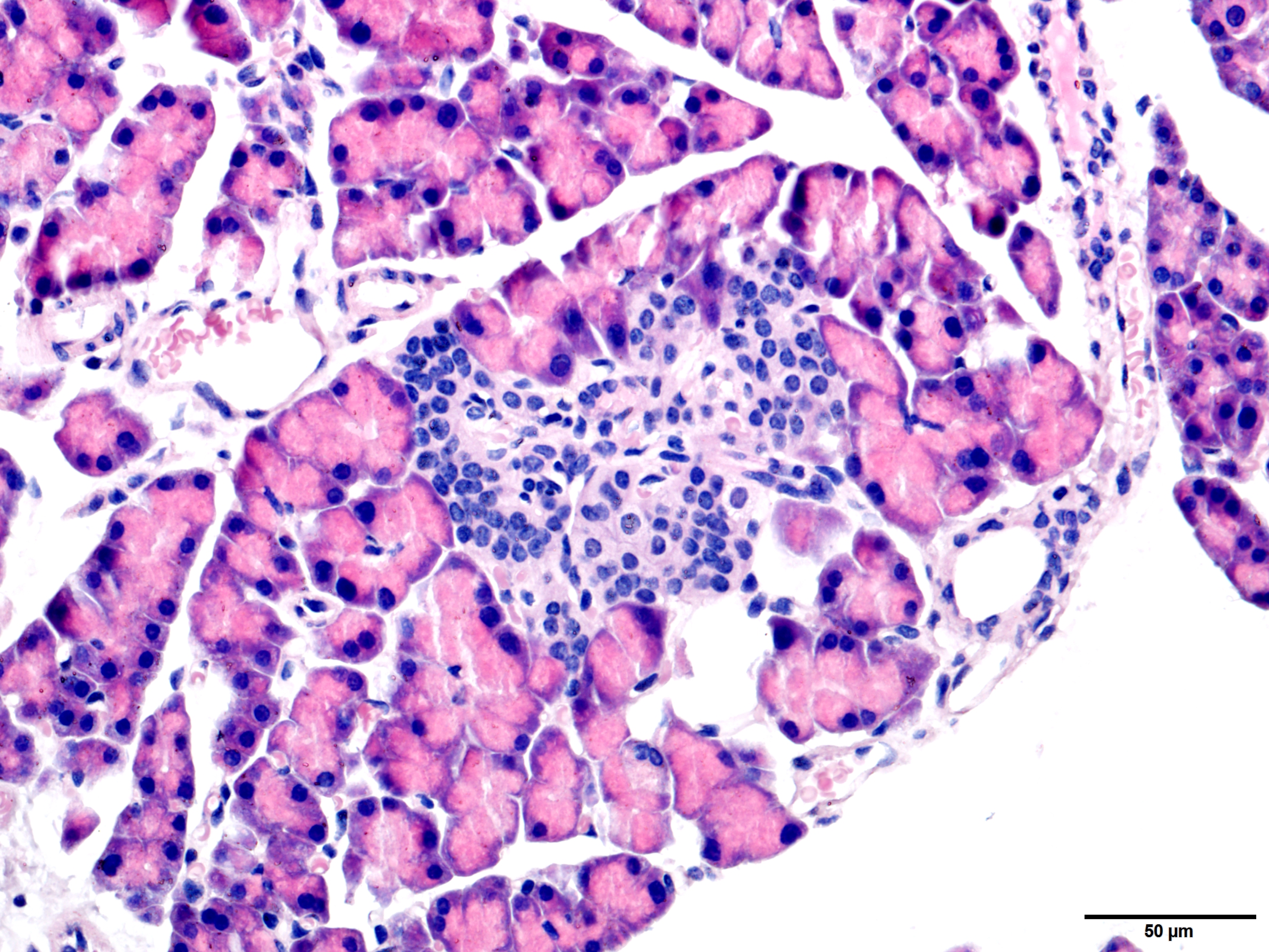

Supplement: Supplemental Information 2 [file peerj-11-15705-s002.zip › Fig.1 Physiology/OA+Etoh.jpg]

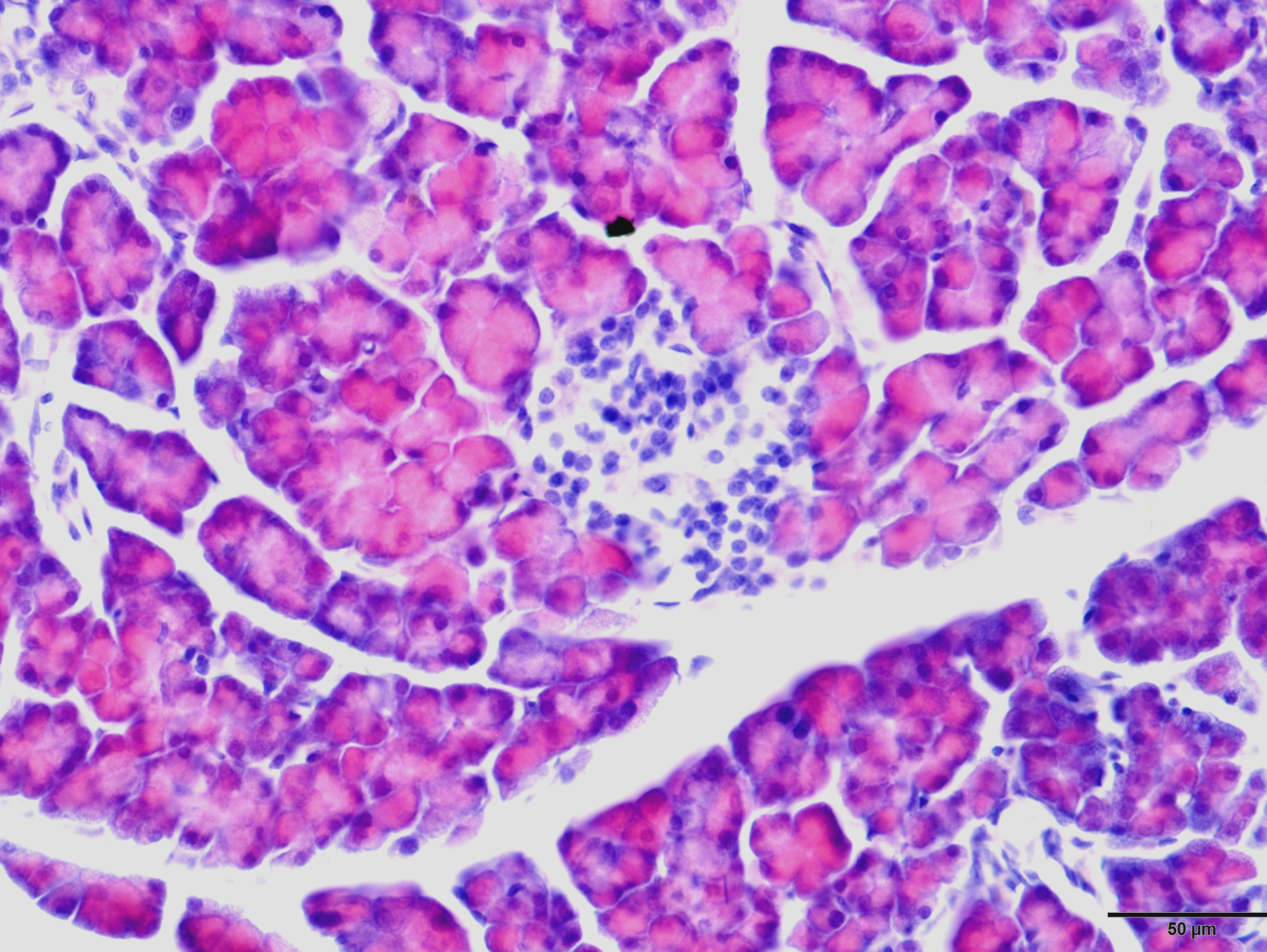

Supplement: Supplemental Information 2 [file peerj-11-15705-s002.zip › Fig.1 Physiology/OA.jpg]

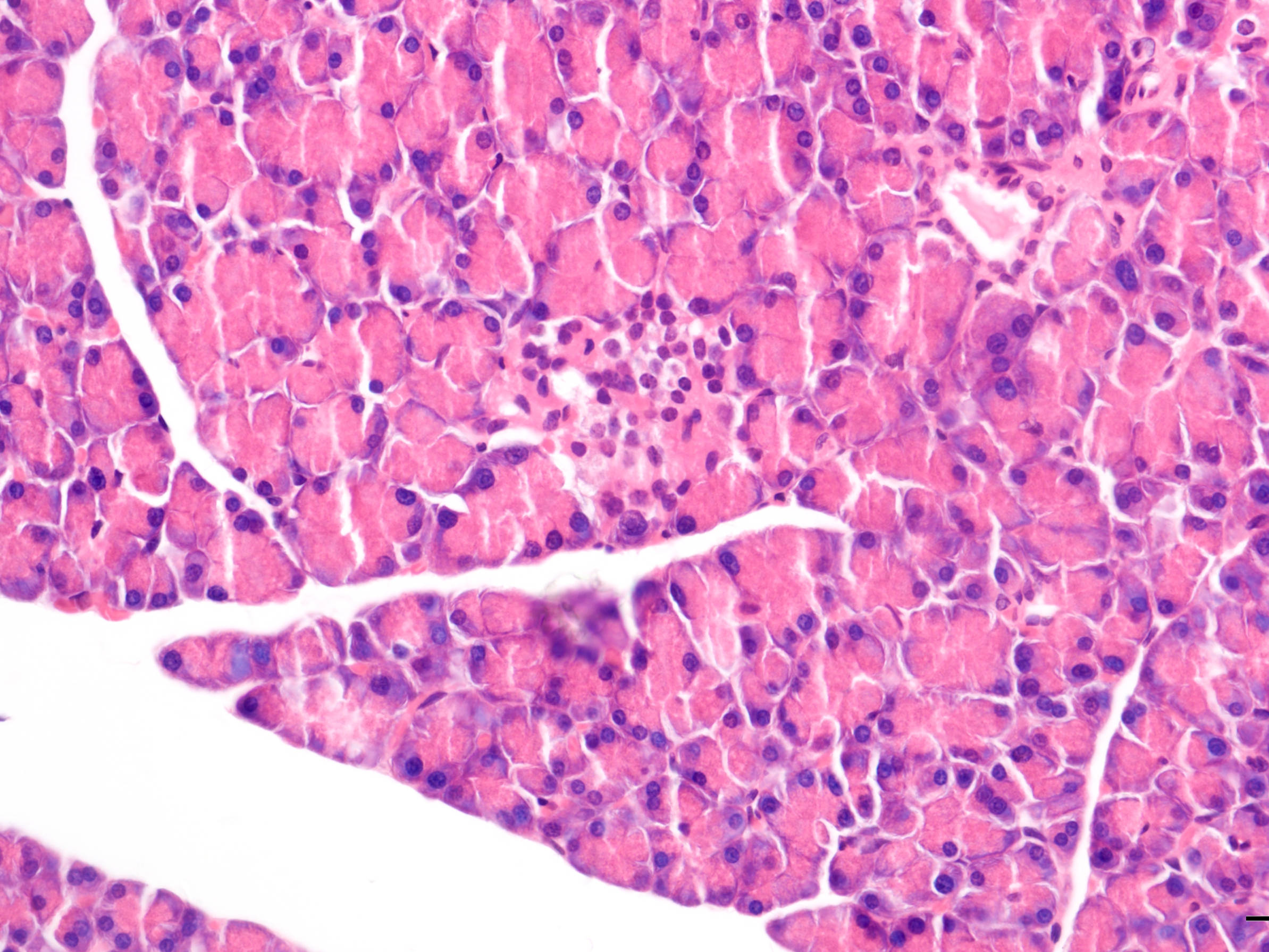

Supplement: Supplemental Information 2 [file peerj-11-15705-s002.zip › Fig.1 Physiology/STZ.jpg]

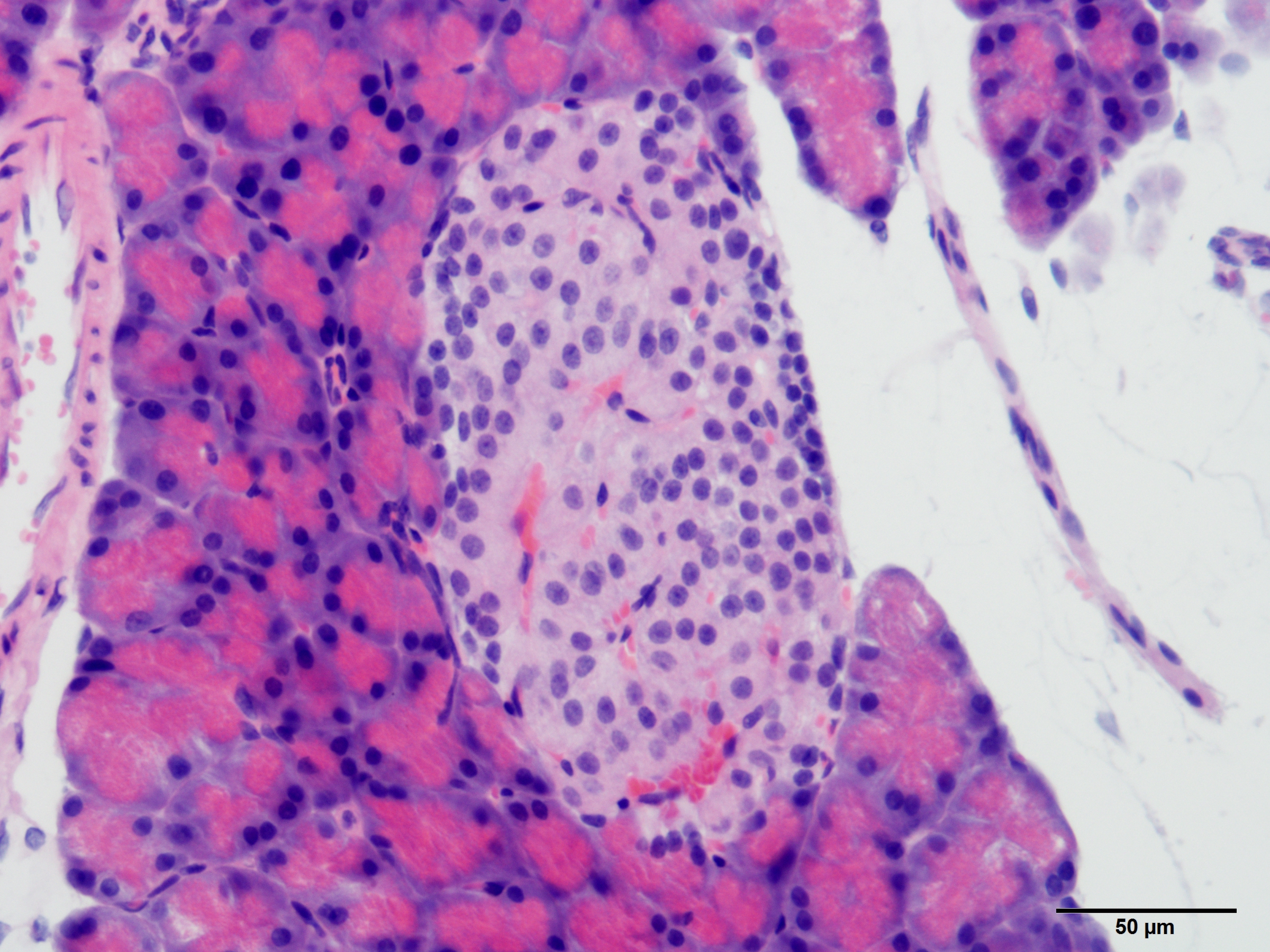

Supplement: Supplemental Information 2 [file peerj-11-15705-s002.zip › Fig.1 Physiology/control.jpg]

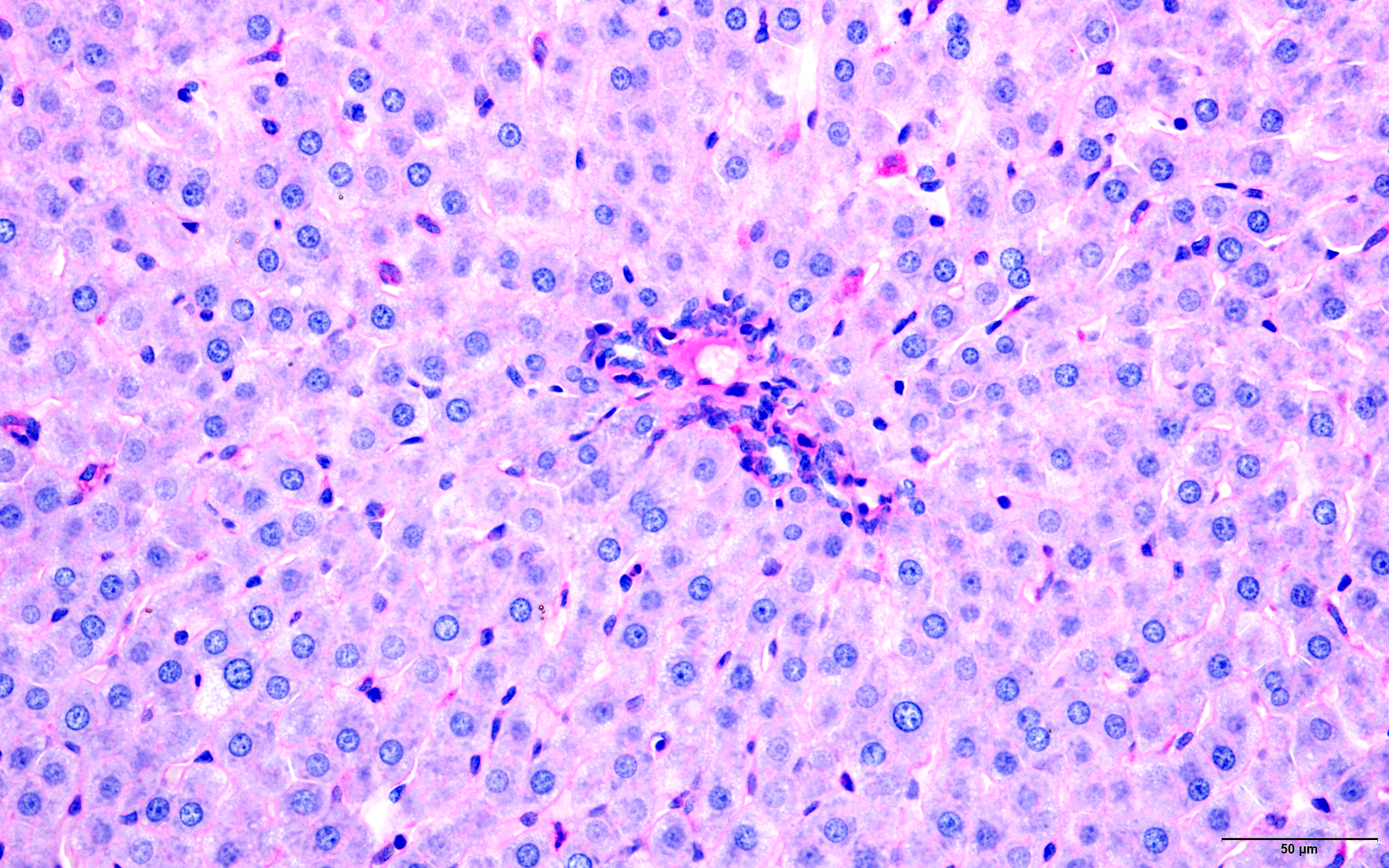

Supplement: Supplemental Information 3 [file peerj-11-15705-s003.zip › Fig.2 PAS Control 400x.tif]

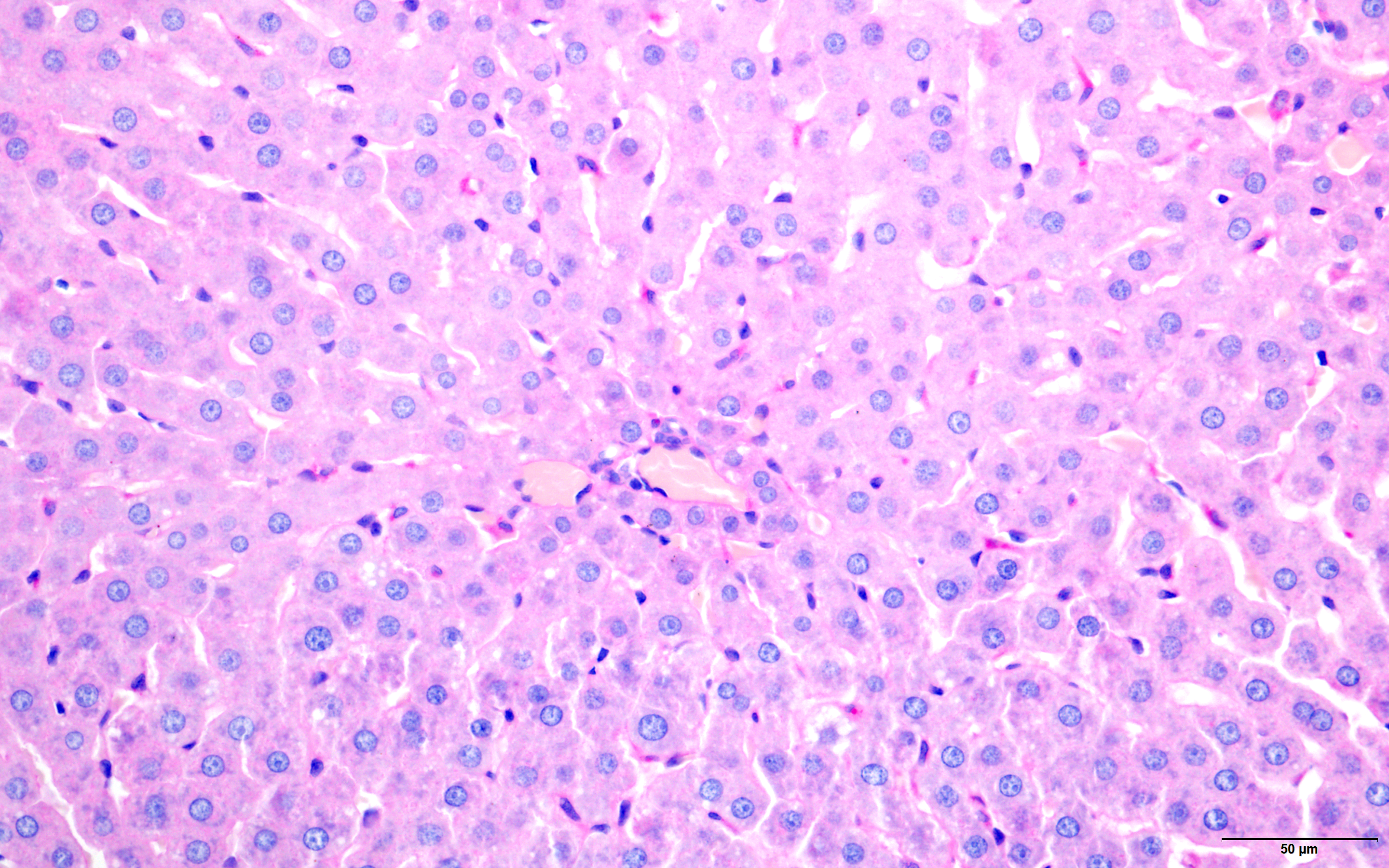

Supplement: Supplemental Information 4 [file peerj-11-15705-s004.zip › Fig.2 PAS OA+E 400x.tif]

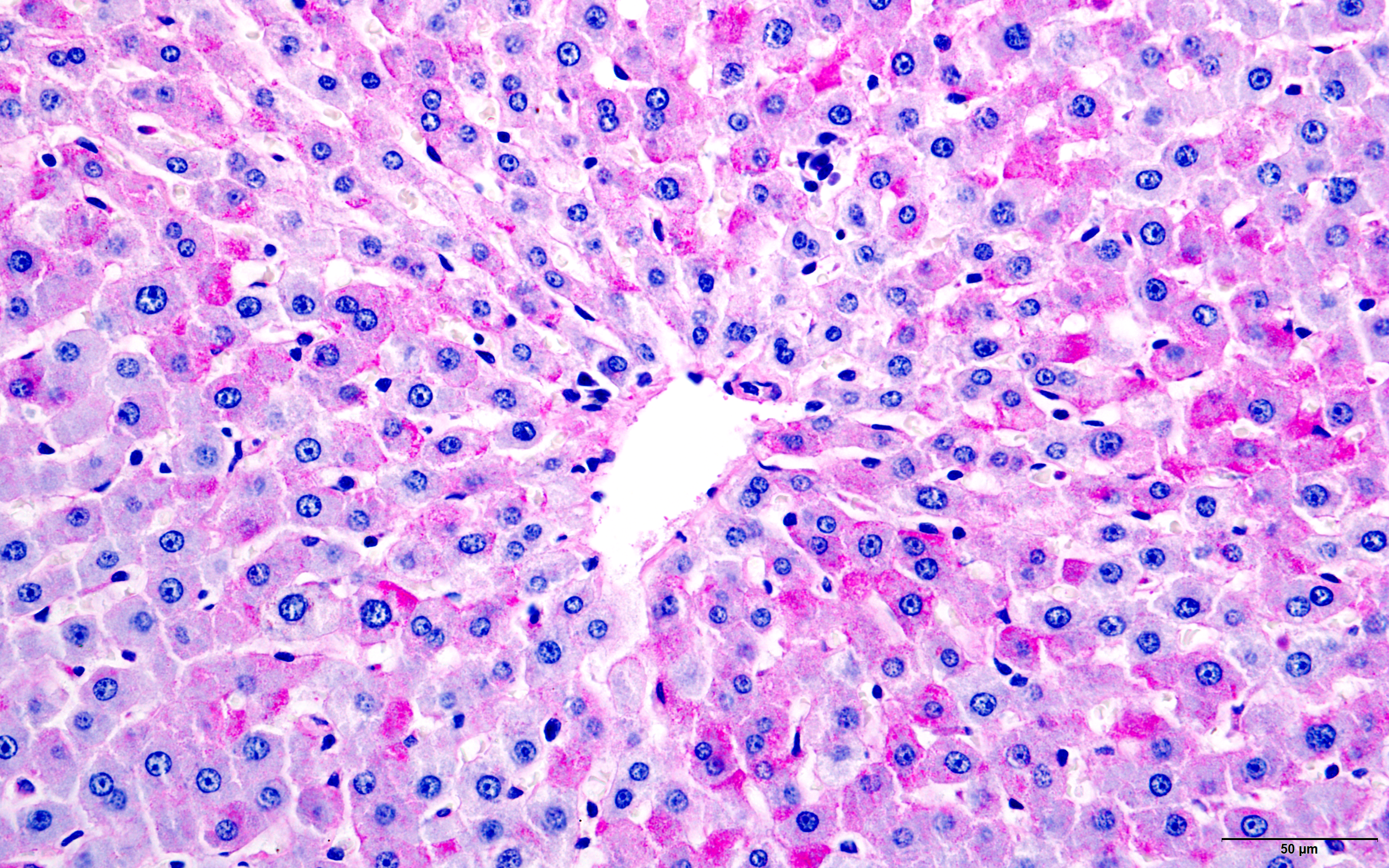

Supplement: Supplemental Information 4 [file peerj-11-15705-s004.zip › Fig.2 PAS EtOH 400x.tif]

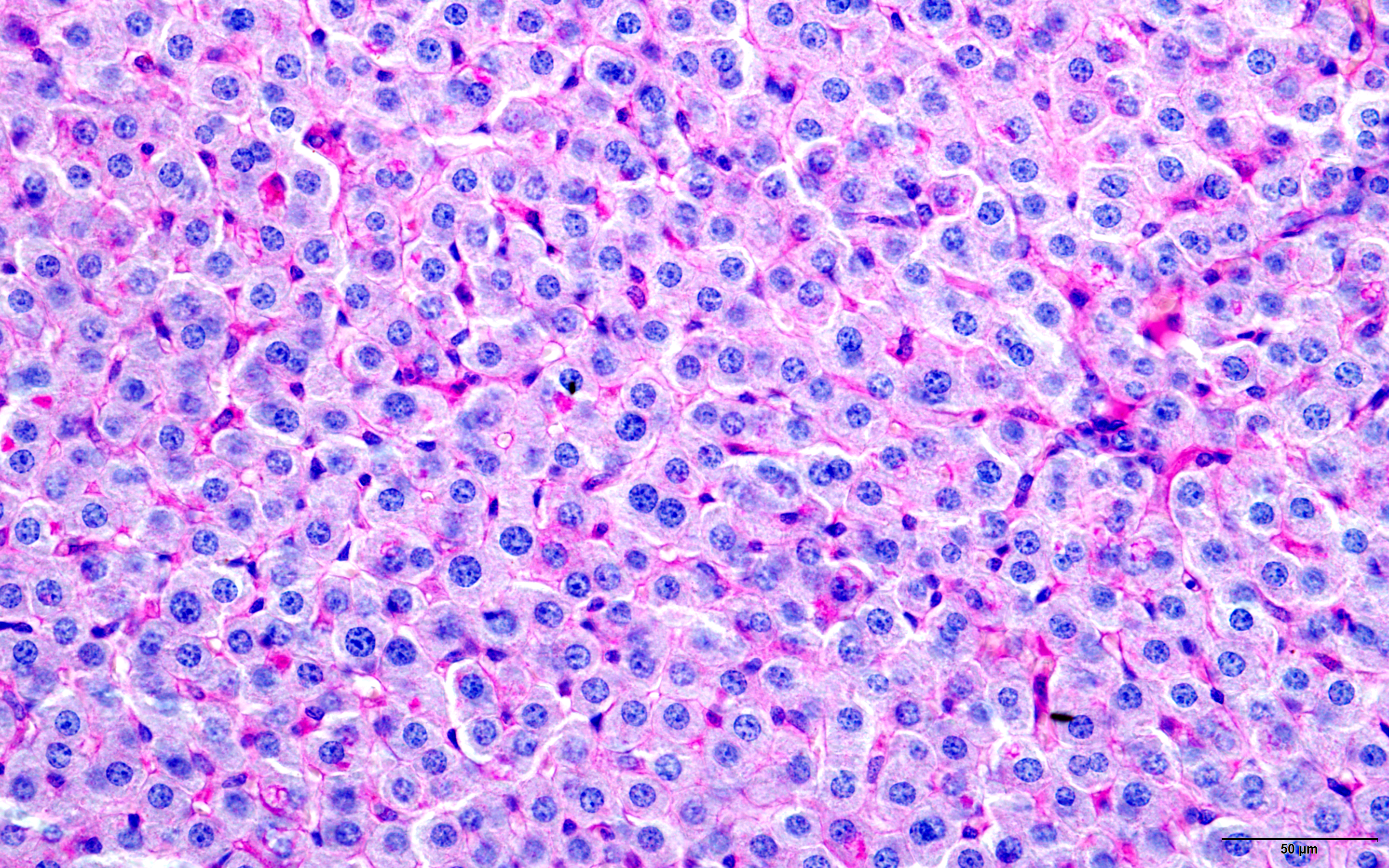

Supplement: Supplemental Information 5 [file peerj-11-15705-s005.zip › Fig.2 PAS STZ 400x.tif]

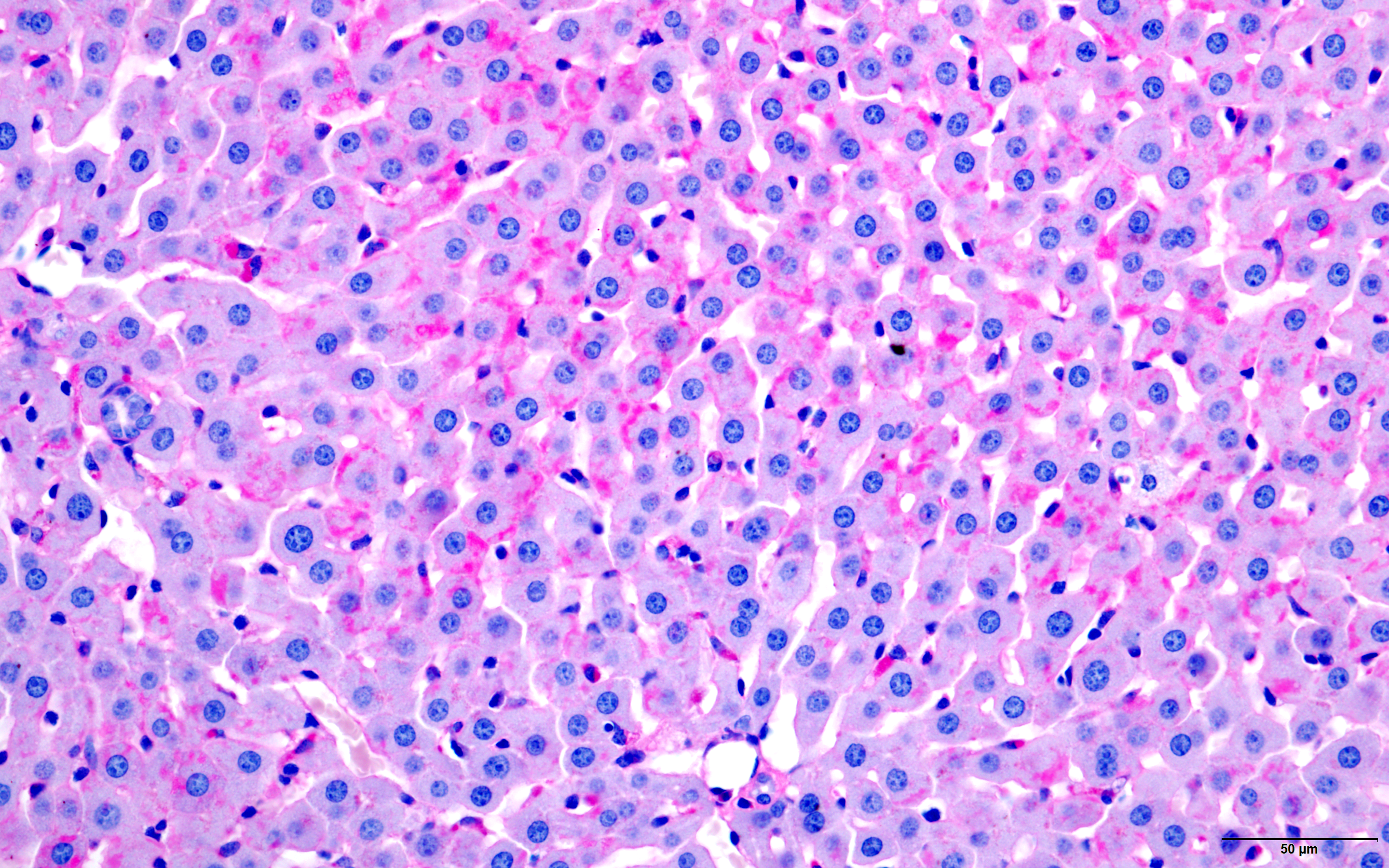

Supplement: Supplemental Information 5 [file peerj-11-15705-s005.zip › Fig.2 PAS OA 400x.tif]

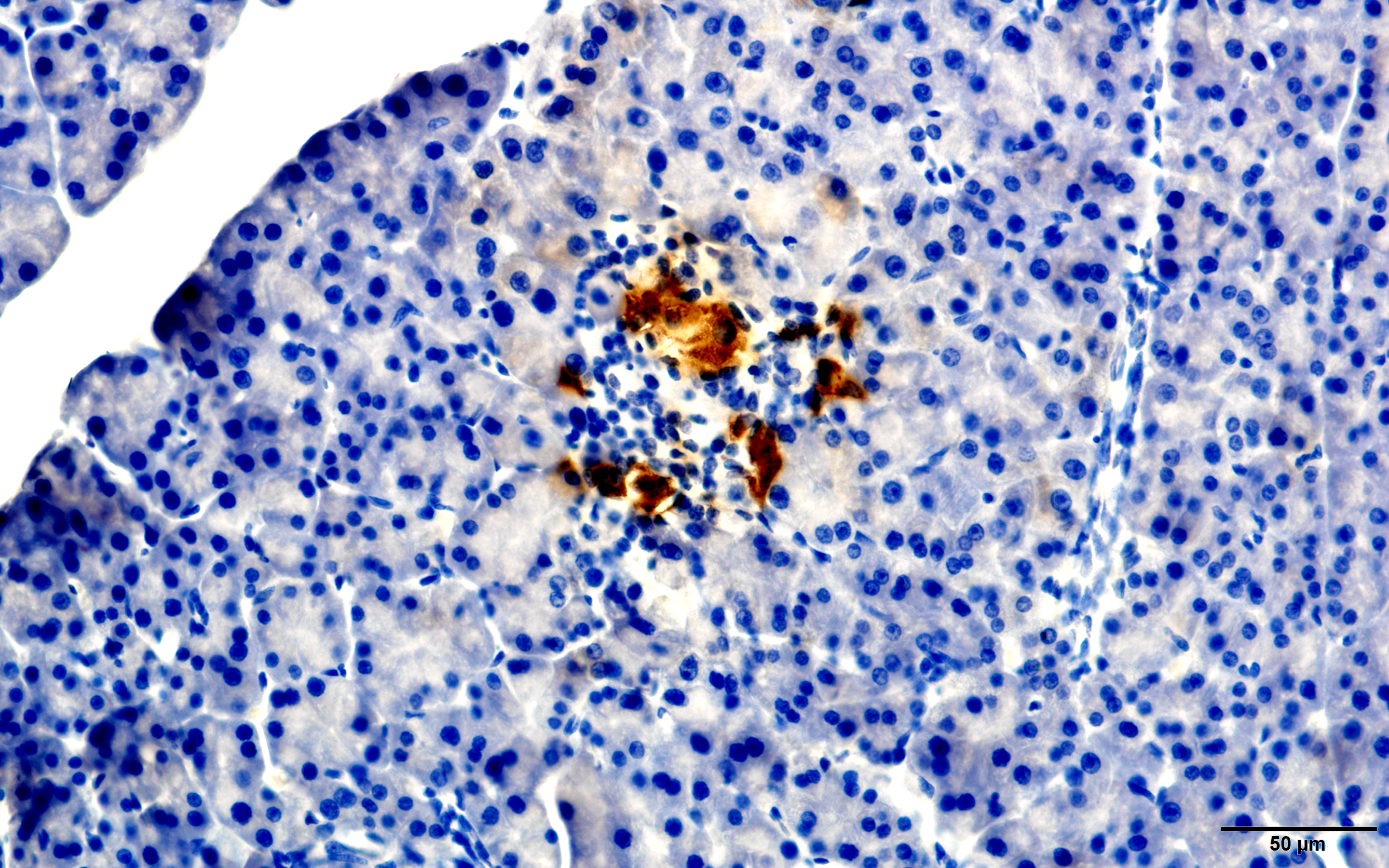

Supplement: Supplemental Information 6 [file peerj-11-15705-s006.zip › Fig.3STZ.tif]

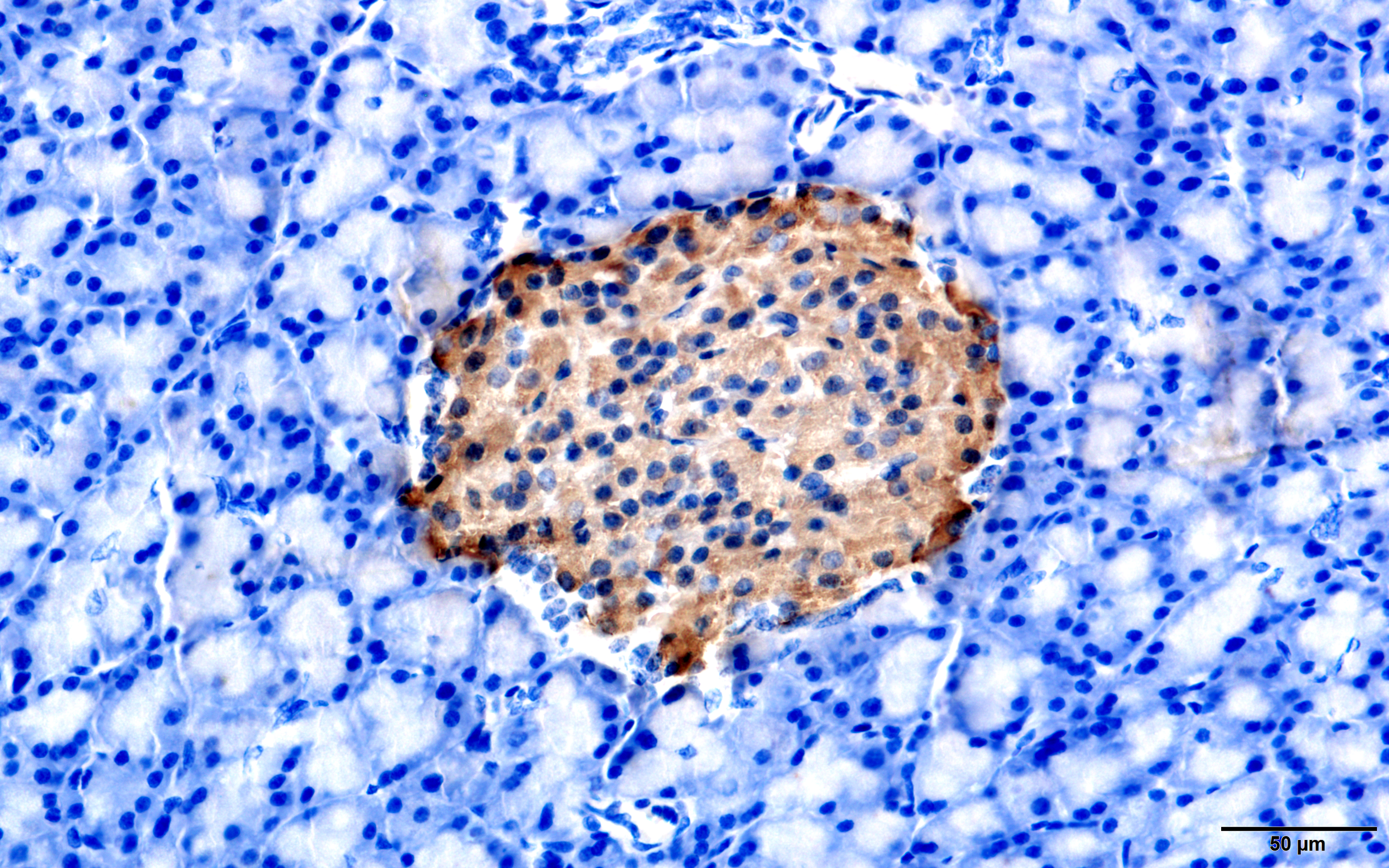

Supplement: Supplemental Information 6 [file peerj-11-15705-s006.zip › Fig.3 Control.tif]

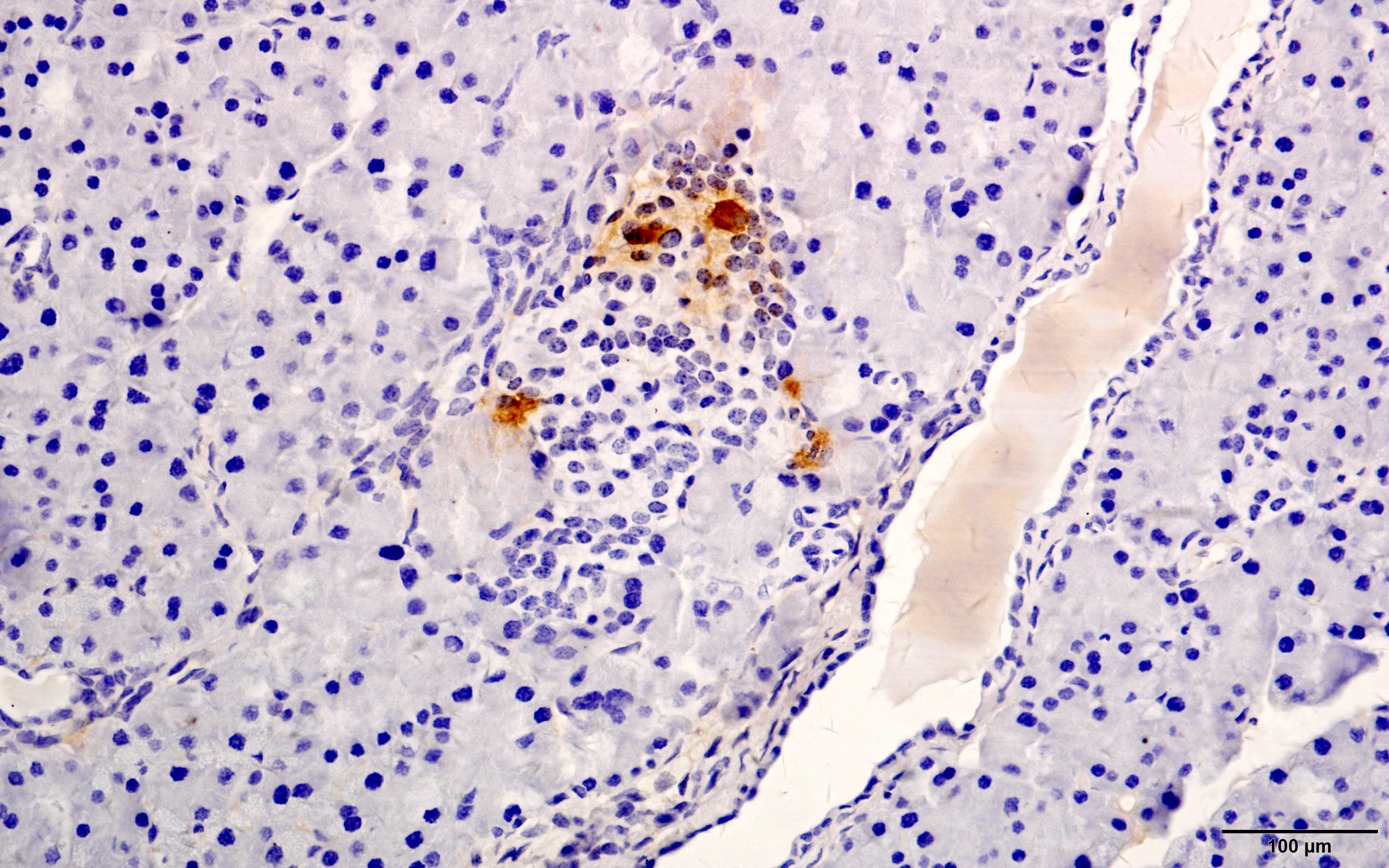

Supplement: Supplemental Information 7 [file peerj-11-15705-s007.zip › Fig.3 OA+E.tif]

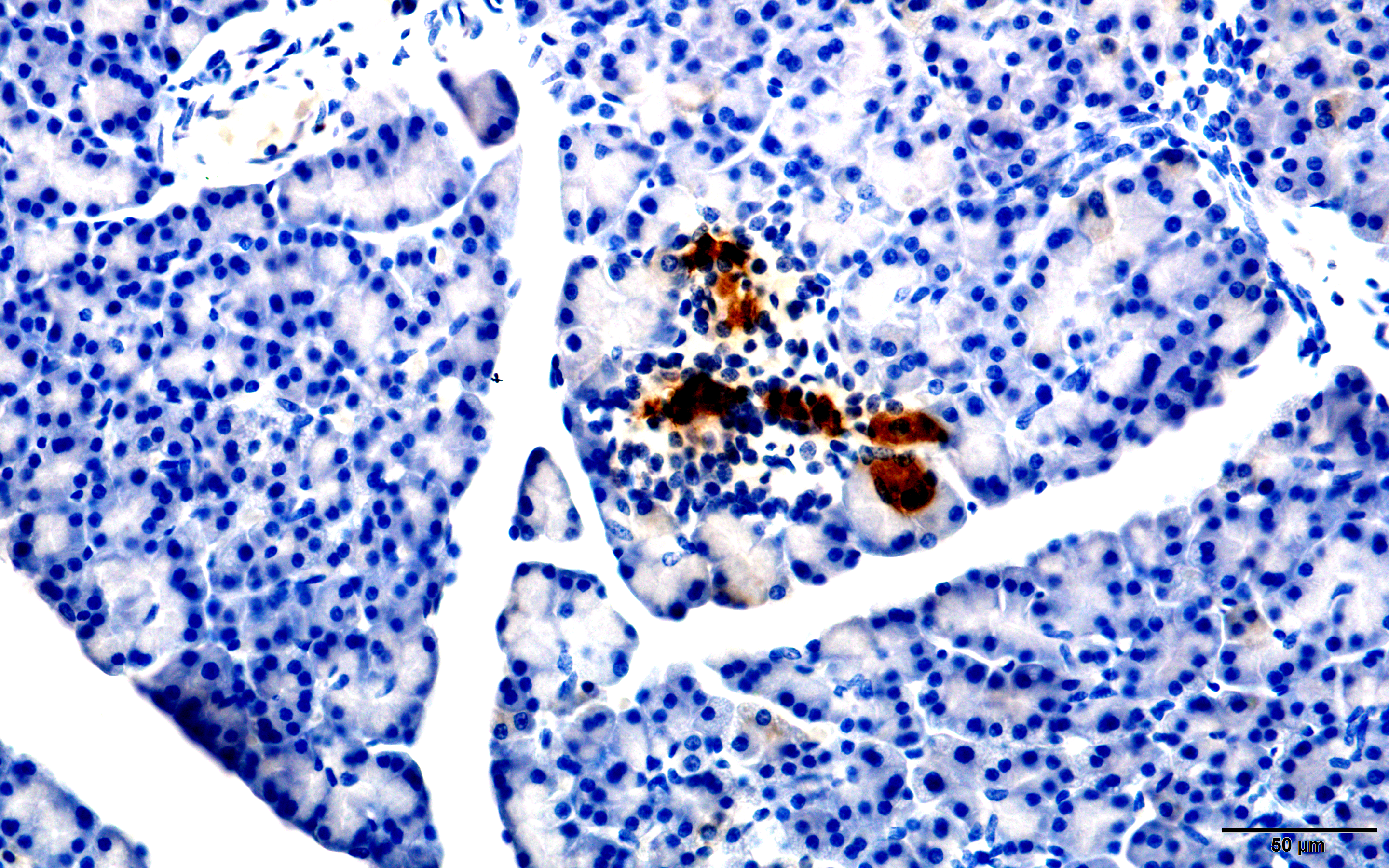

Supplement: Supplemental Information 7 [file peerj-11-15705-s007.zip › Fig.3 OA.tif]

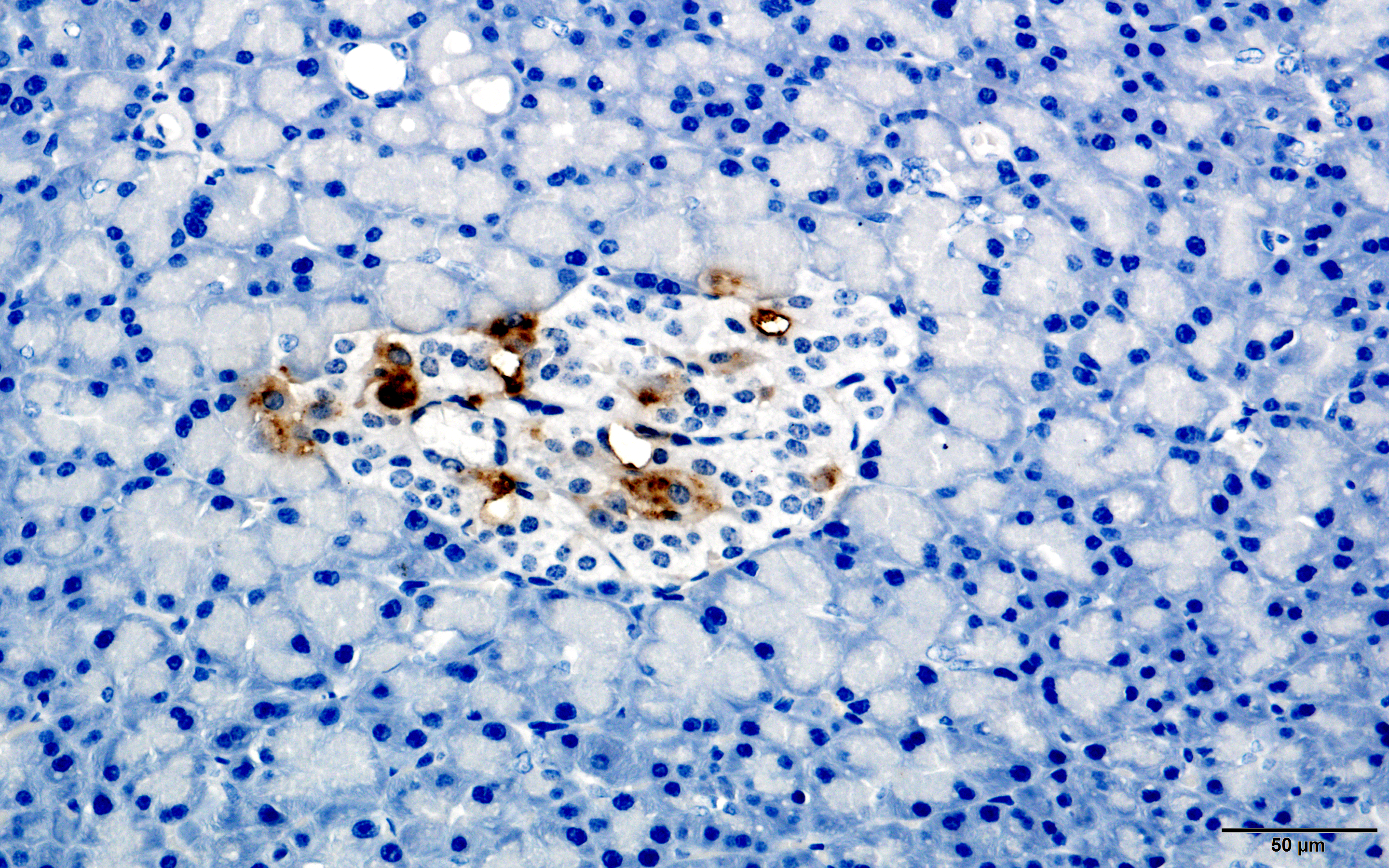

Supplement: Supplemental Information 7 [file peerj-11-15705-s007.zip › Fig.3 EtOH.tif]

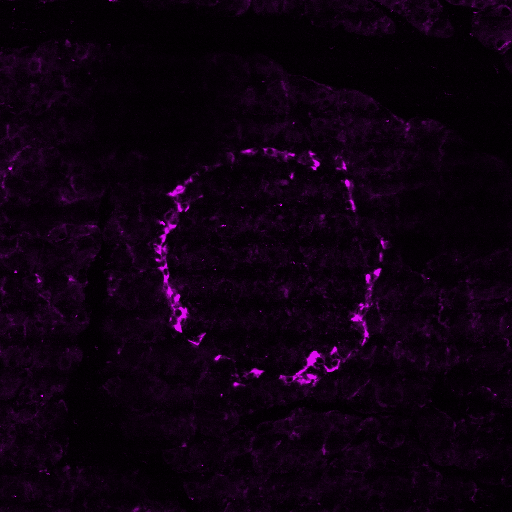

Supplement: Supplemental Information 8 [file peerj-11-15705-s008.zip › Fig.4 INS and GLU/Control/Gcg.tif]

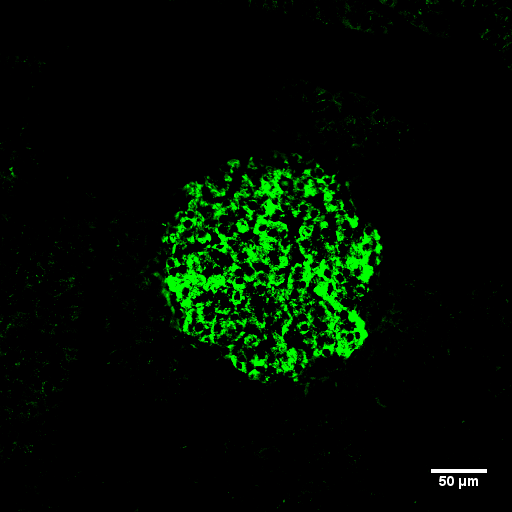

Supplement: Supplemental Information 8 [file peerj-11-15705-s008.zip › Fig.4 INS and GLU/Control/Ins.tif]

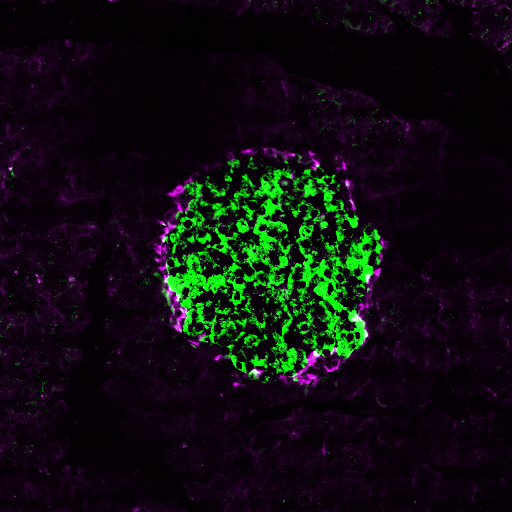

Supplement: Supplemental Information 8 [file peerj-11-15705-s008.zip › Fig.4 INS and GLU/Control/merge.tif]

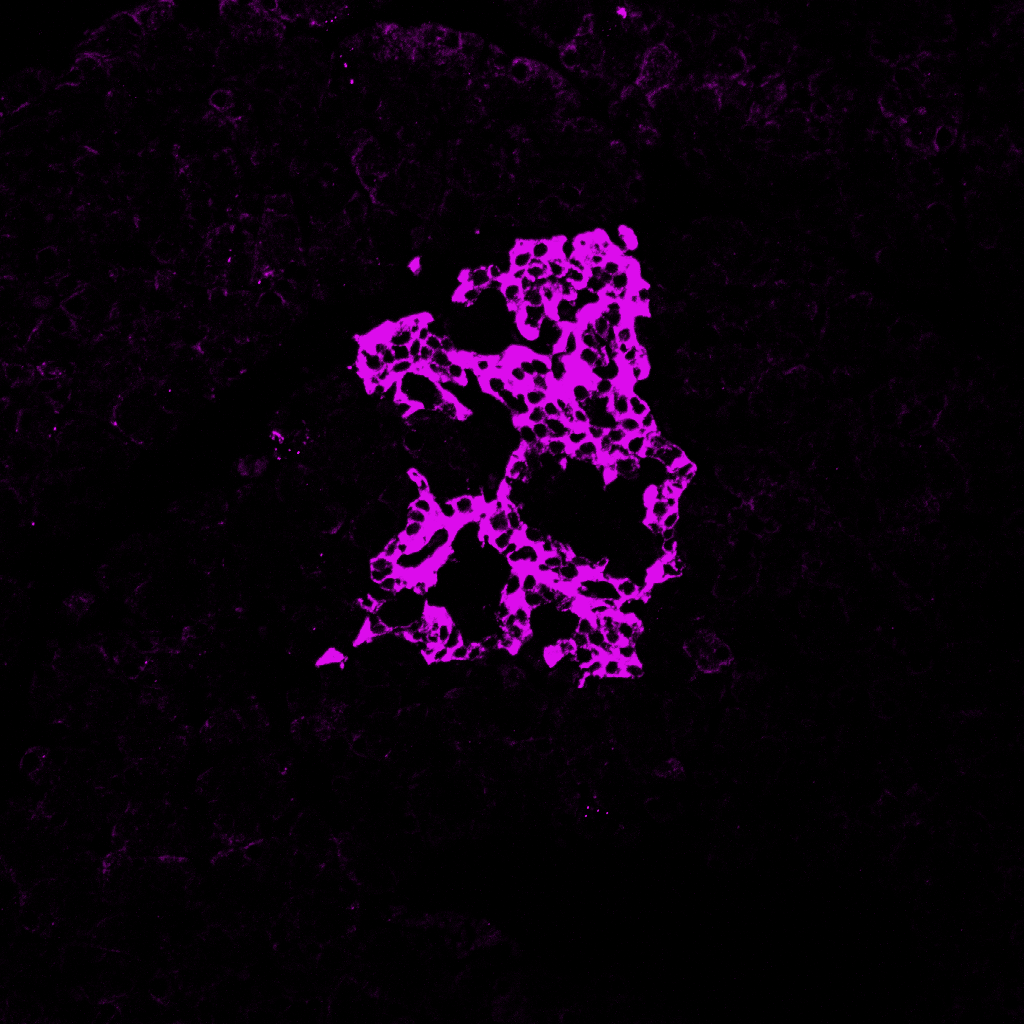

Supplement: Supplemental Information 8 [file peerj-11-15705-s008.zip › Fig.4 INS and GLU/EtOH/Gcg.tif]

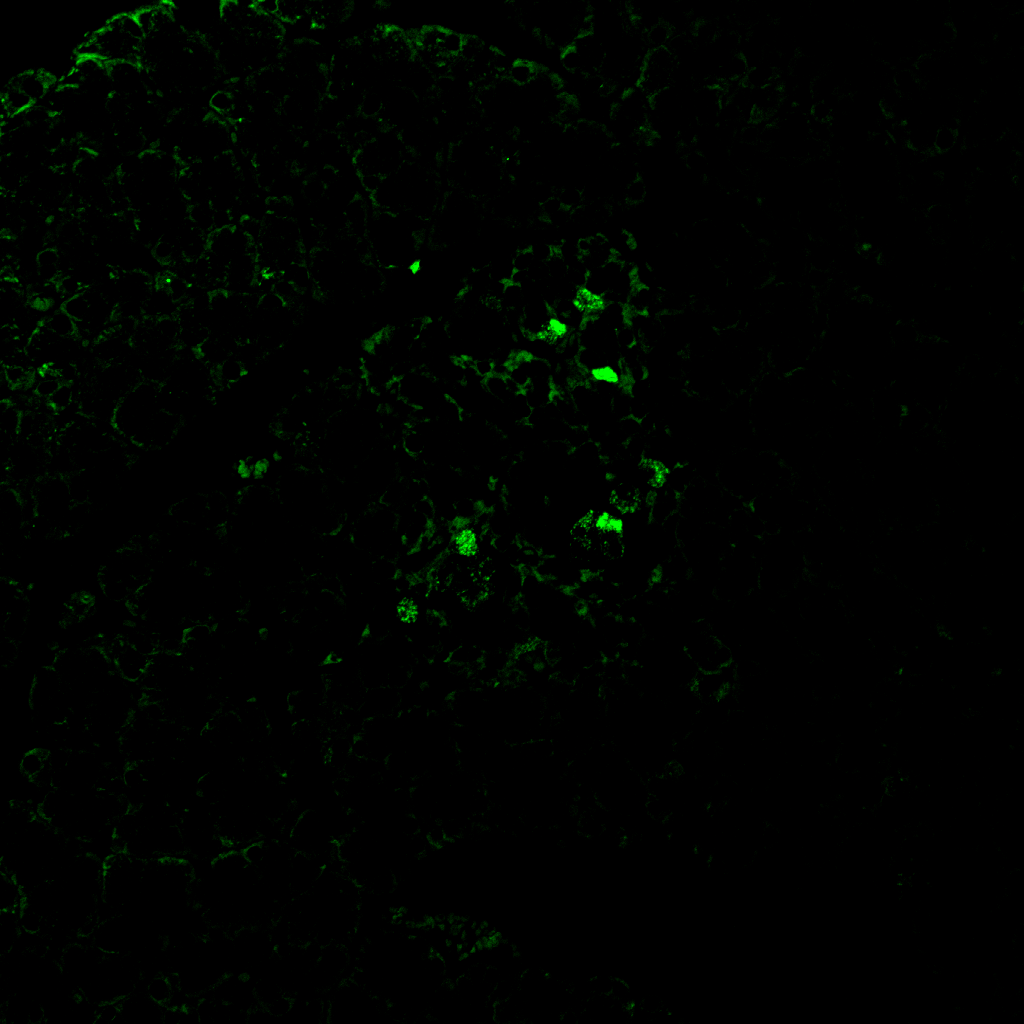

Supplement: Supplemental Information 8 [file peerj-11-15705-s008.zip › Fig.4 INS and GLU/EtOH/Ins.tif]

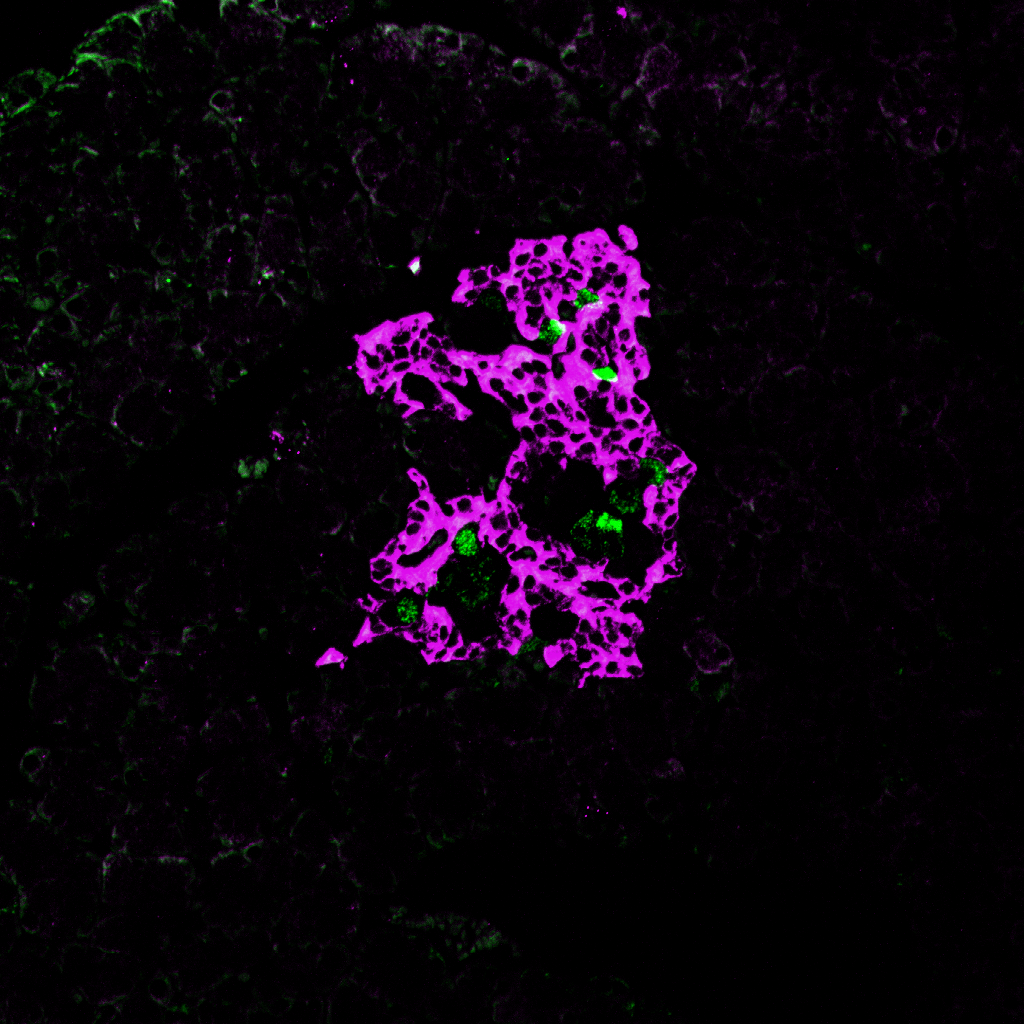

Supplement: Supplemental Information 8 [file peerj-11-15705-s008.zip › Fig.4 INS and GLU/EtOH/merge.tif]

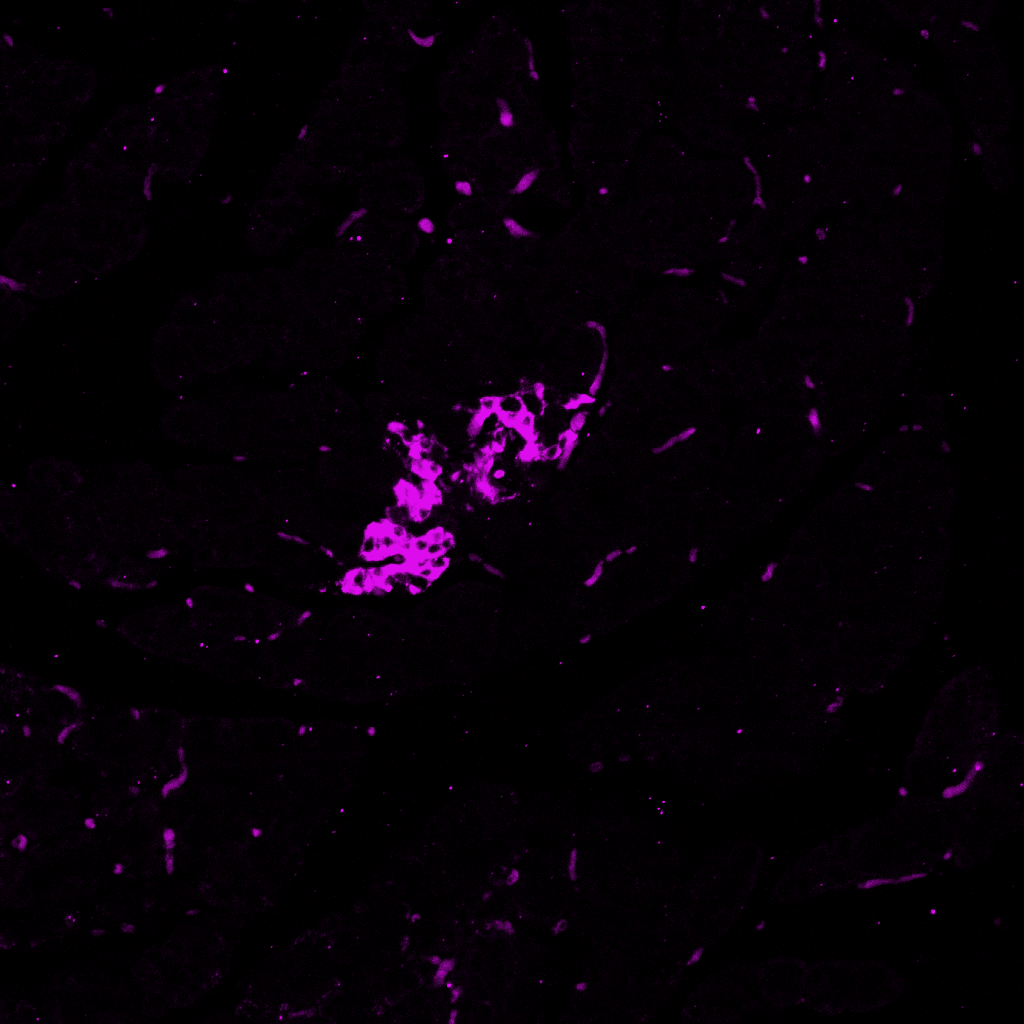

Supplement: Supplemental Information 8 [file peerj-11-15705-s008.zip › Fig.4 INS and GLU/OA/Gcg.tif]

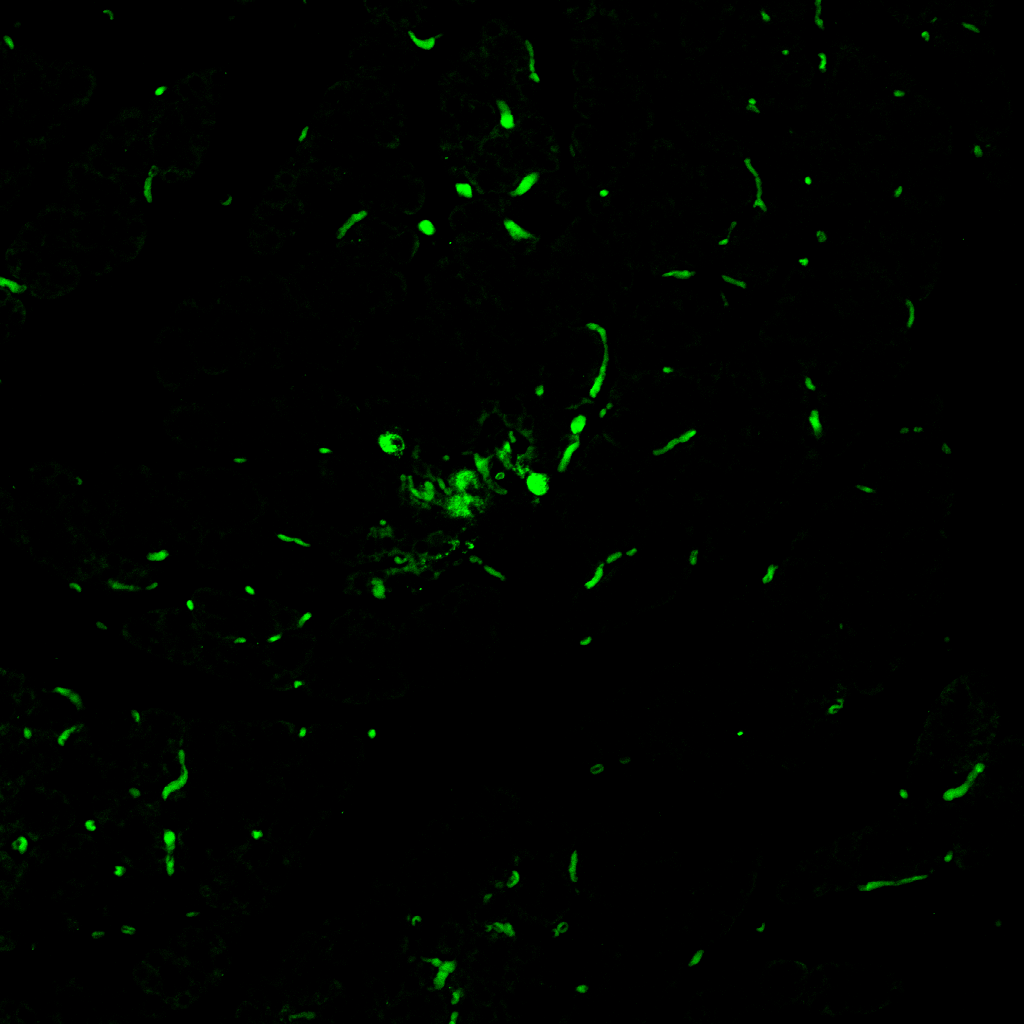

Supplement: Supplemental Information 8 [file peerj-11-15705-s008.zip › Fig.4 INS and GLU/OA/Ins.tif]

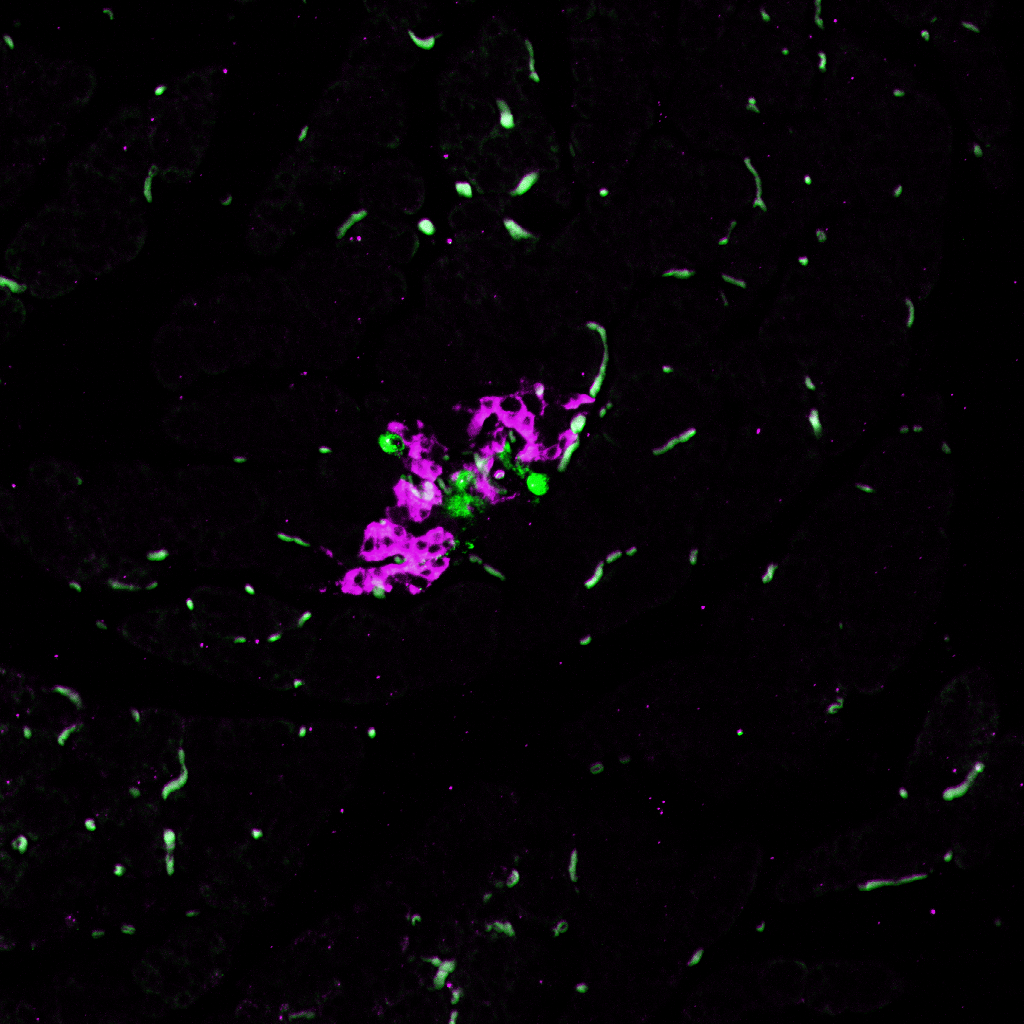

Supplement: Supplemental Information 8 [file peerj-11-15705-s008.zip › Fig.4 INS and GLU/OA/merge.tif]

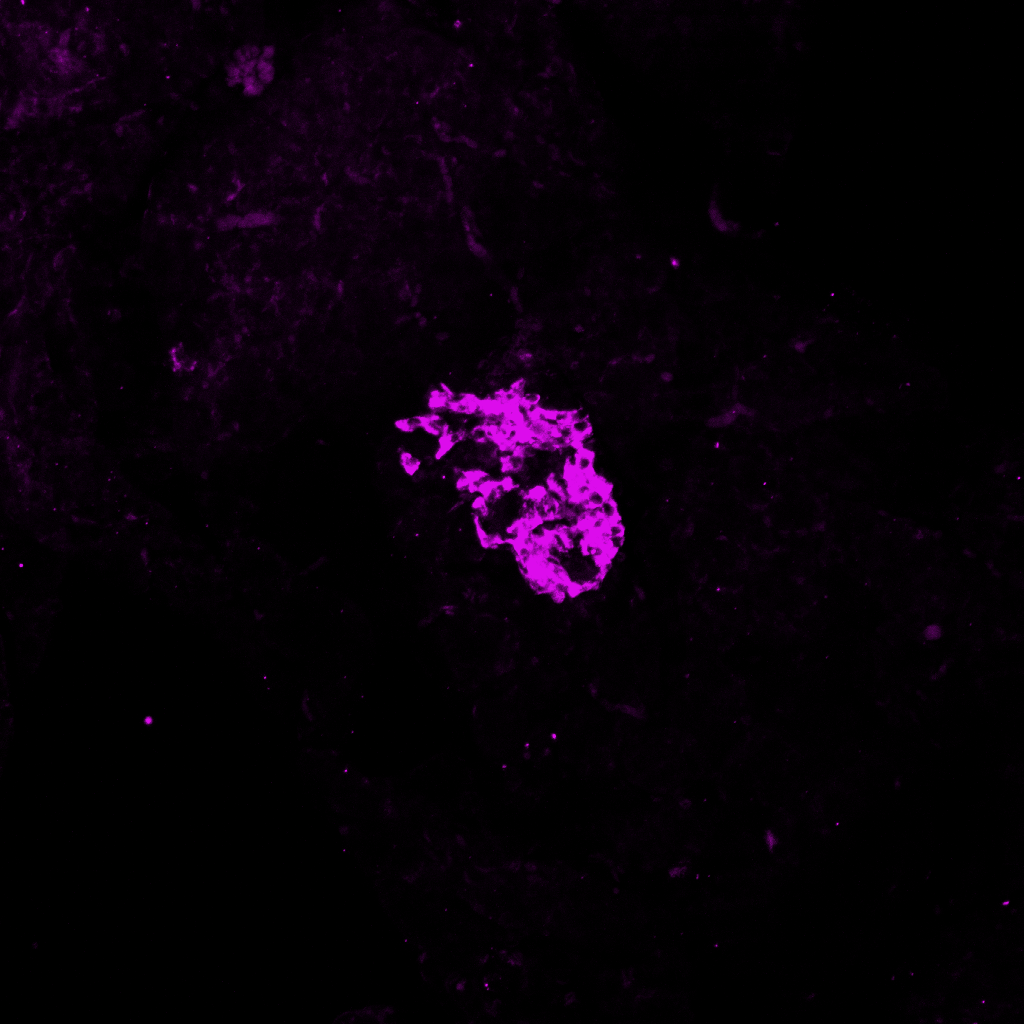

Supplement: Supplemental Information 8 [file peerj-11-15705-s008.zip › Fig.4 INS and GLU/OA+E/Gcg.tif]

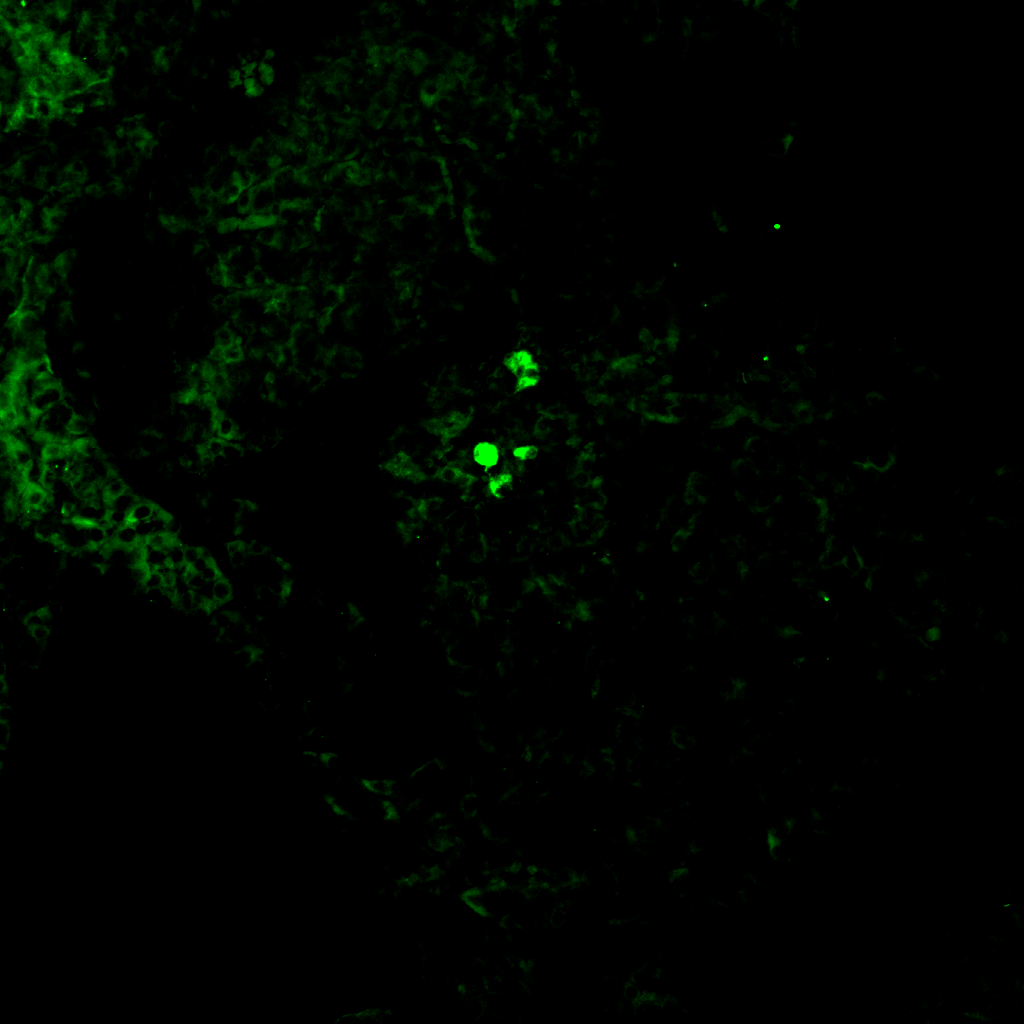

Supplement: Supplemental Information 8 [file peerj-11-15705-s008.zip › Fig.4 INS and GLU/OA+E/Ins.tif]

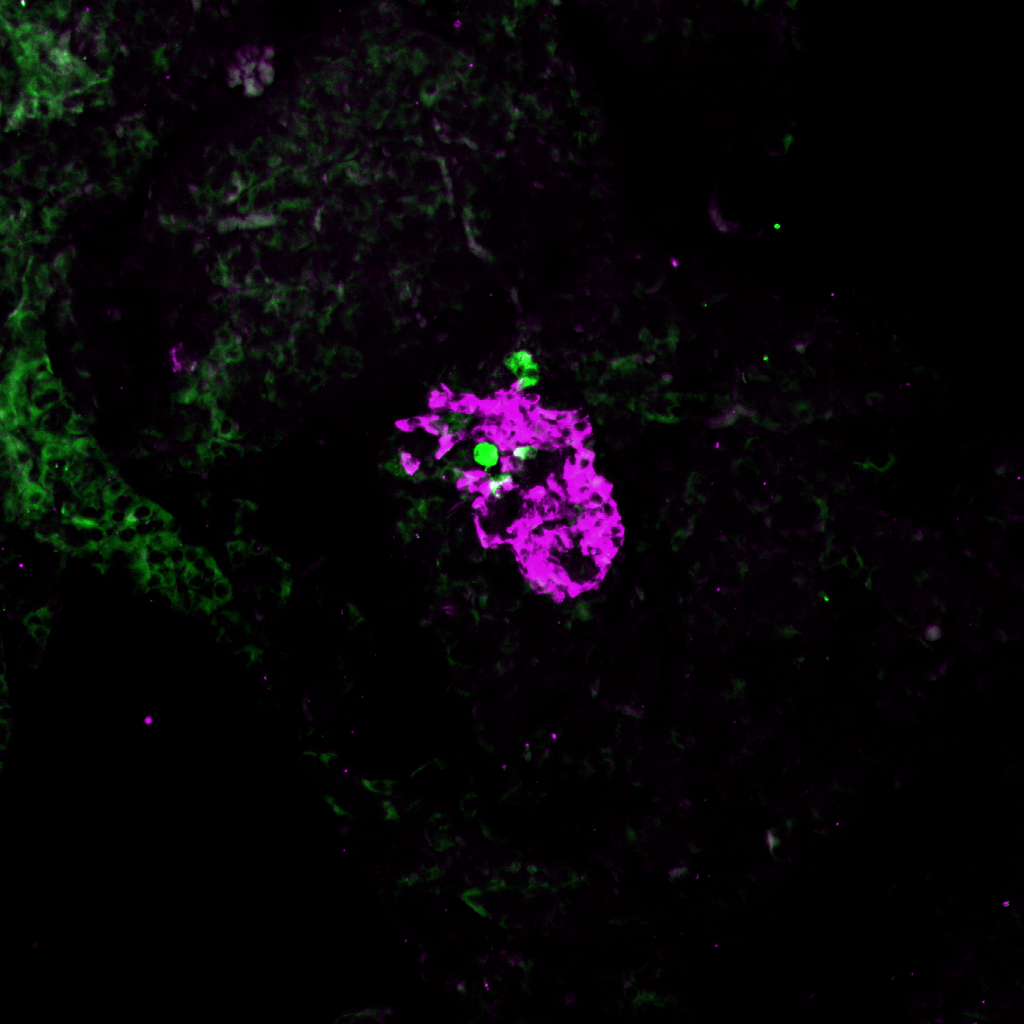

Supplement: Supplemental Information 8 [file peerj-11-15705-s008.zip › Fig.4 INS and GLU/OA+E/merge.tif]

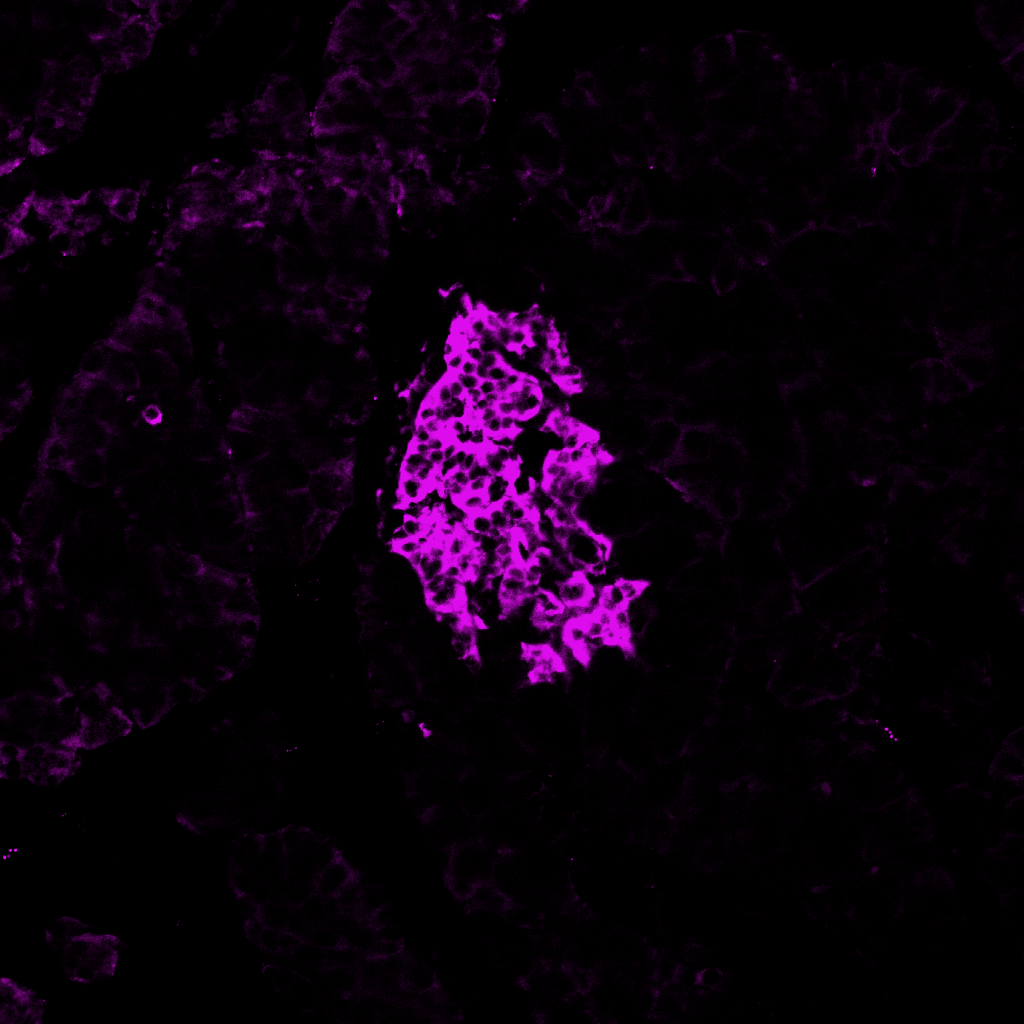

Supplement: Supplemental Information 8 [file peerj-11-15705-s008.zip › Fig.4 INS and GLU/STZ/Gcg.tif]

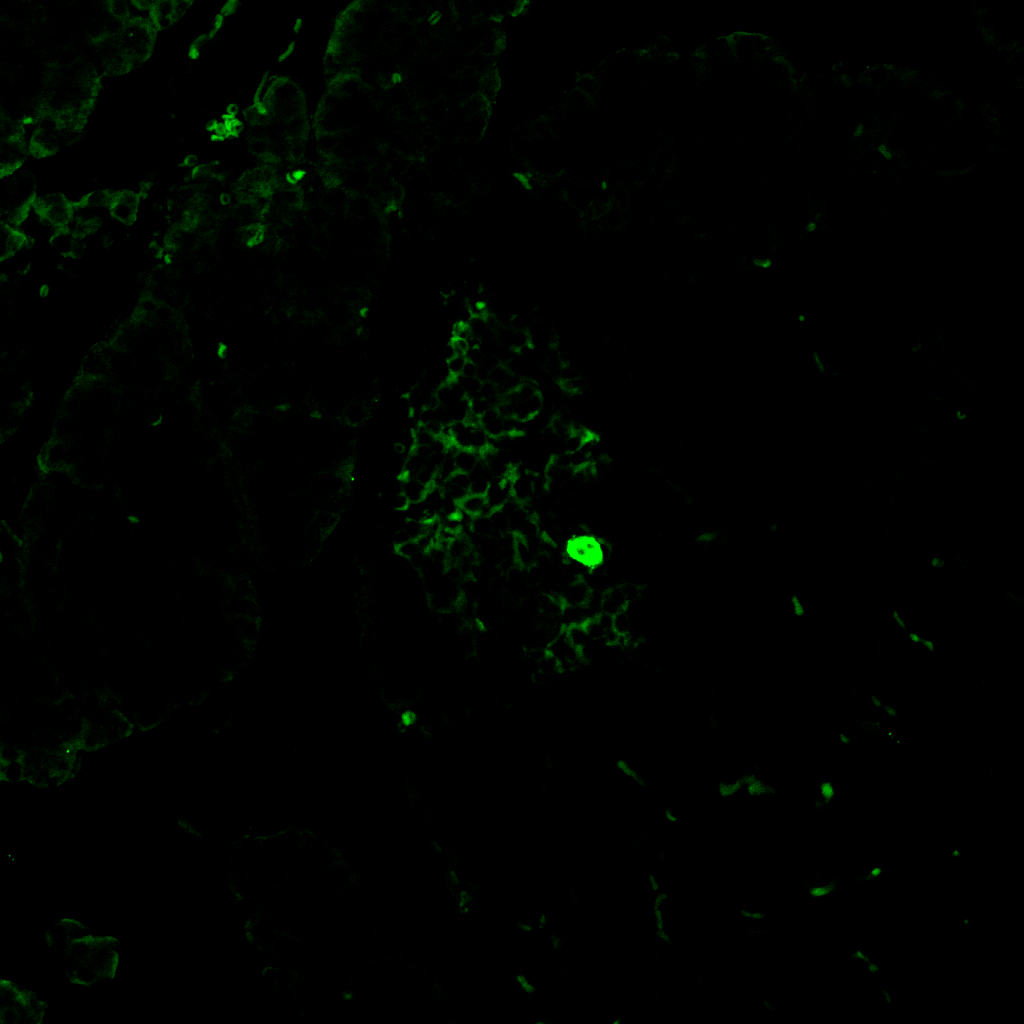

Supplement: Supplemental Information 8 [file peerj-11-15705-s008.zip › Fig.4 INS and GLU/STZ/Ins.tif]

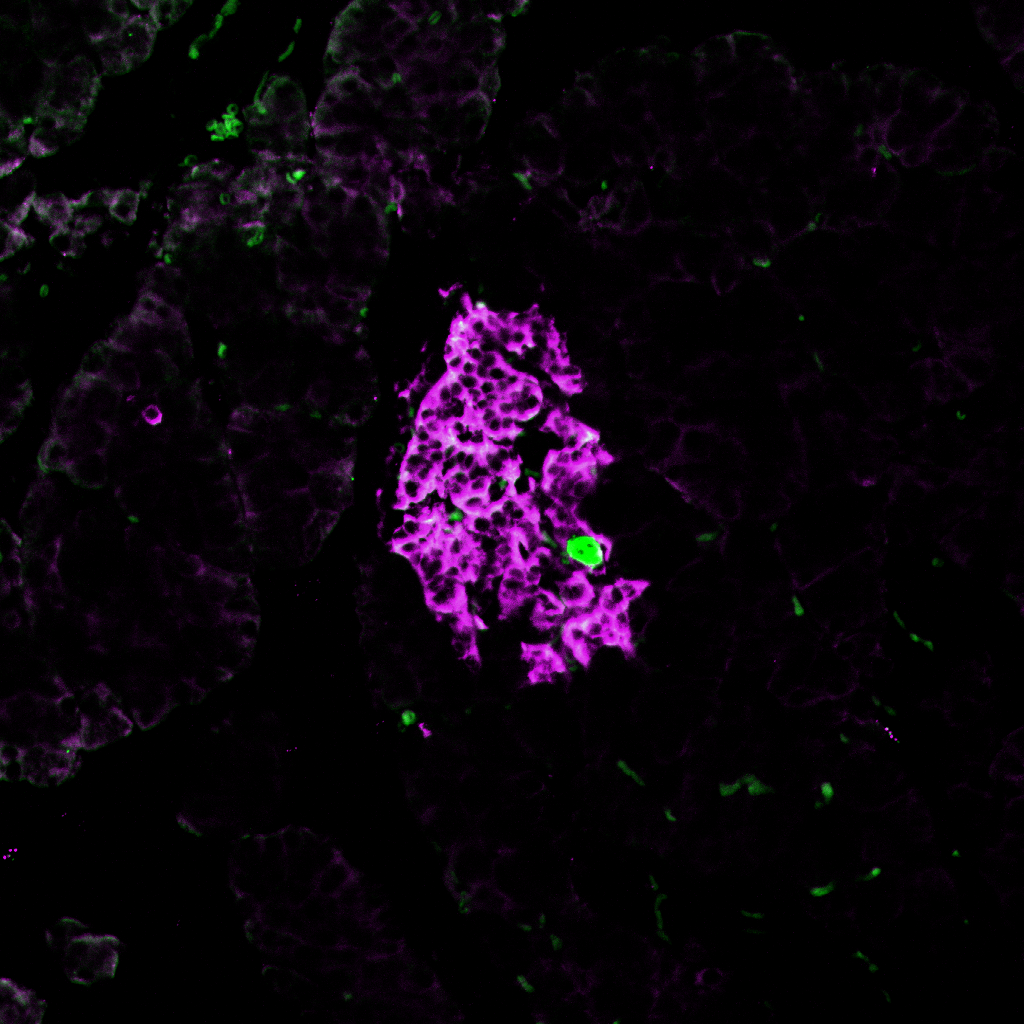

Supplement: Supplemental Information 8 [file peerj-11-15705-s008.zip › Fig.4 INS and GLU/STZ/merge.tif]

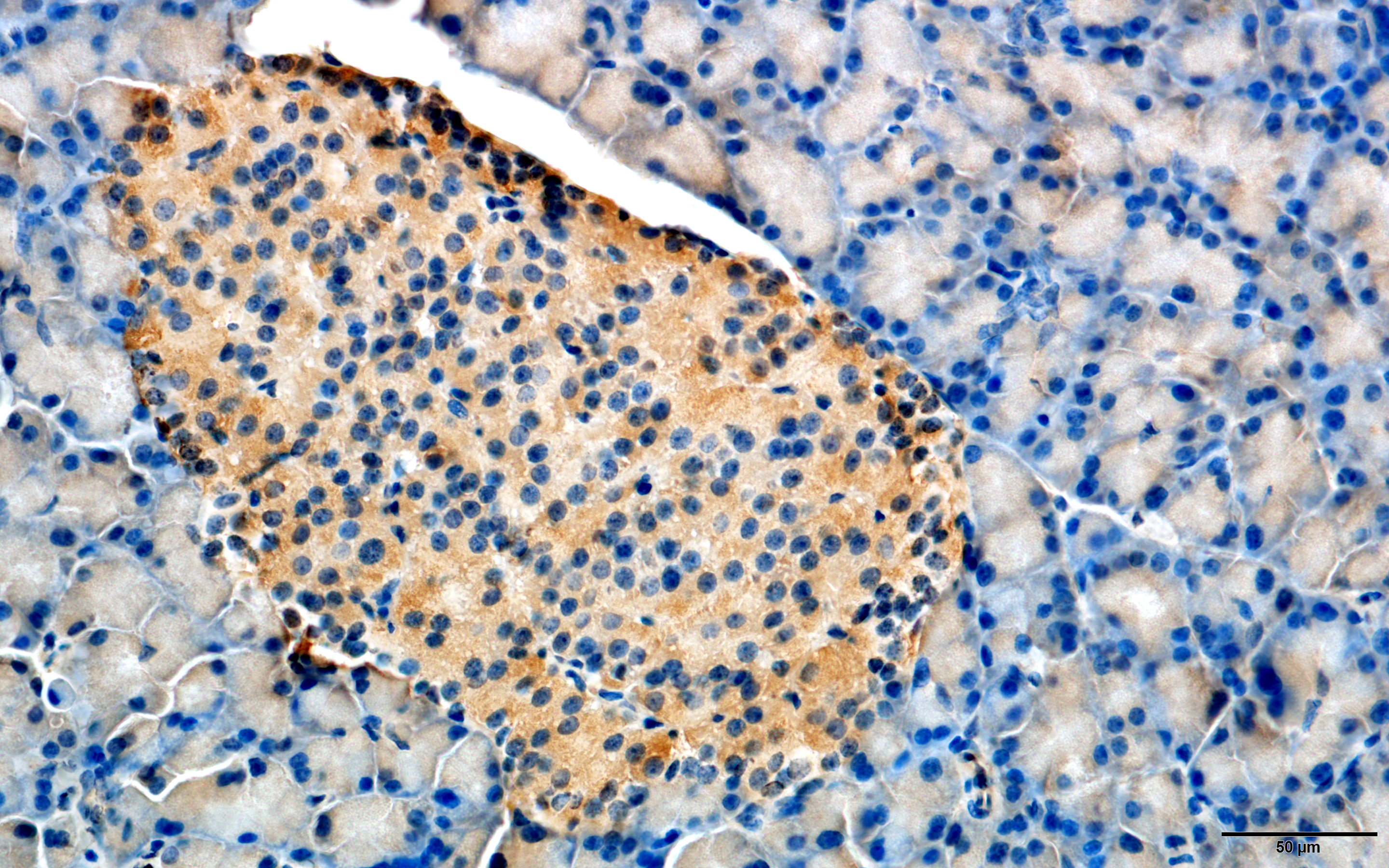

Supplement: Supplemental Information 9 [file peerj-11-15705-s009.zip › Fig.5 Control 400x.tif]

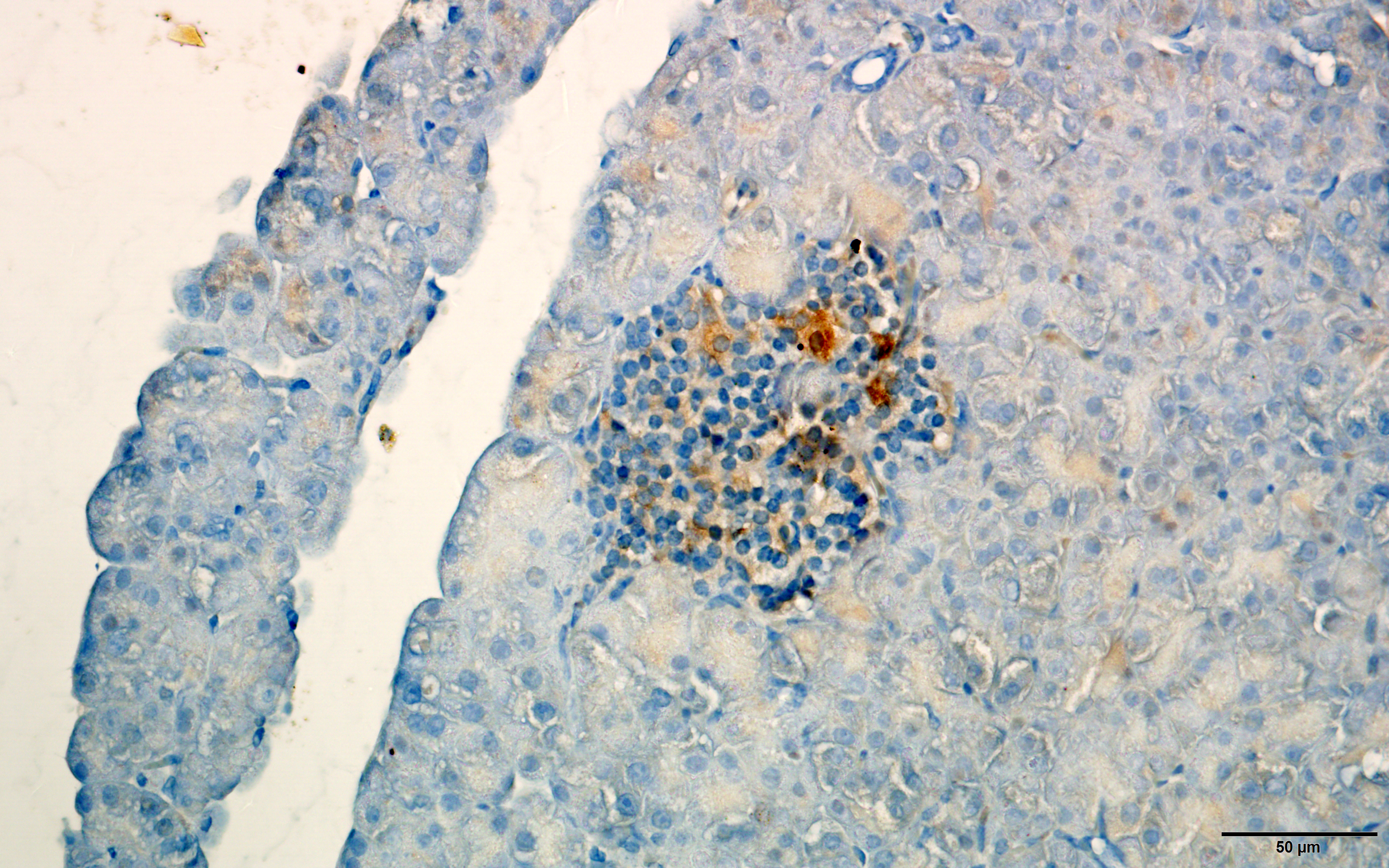

Supplement: Supplemental Information 10 [file peerj-11-15705-s010.zip › Fig.5 OA+E 400x.tif]

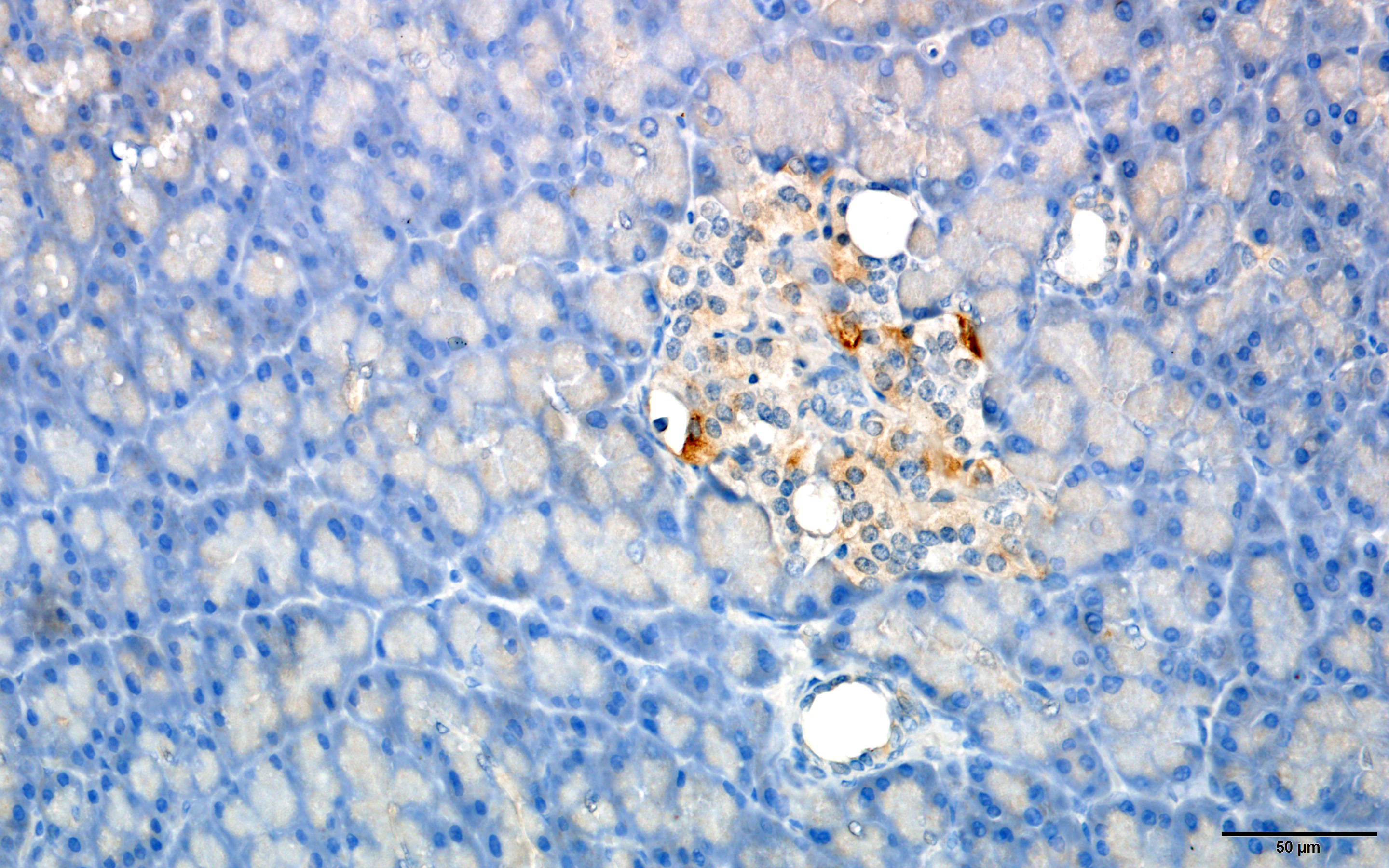

Supplement: Supplemental Information 10 [file peerj-11-15705-s010.zip › Fig.5 EtOH 400x.tif]

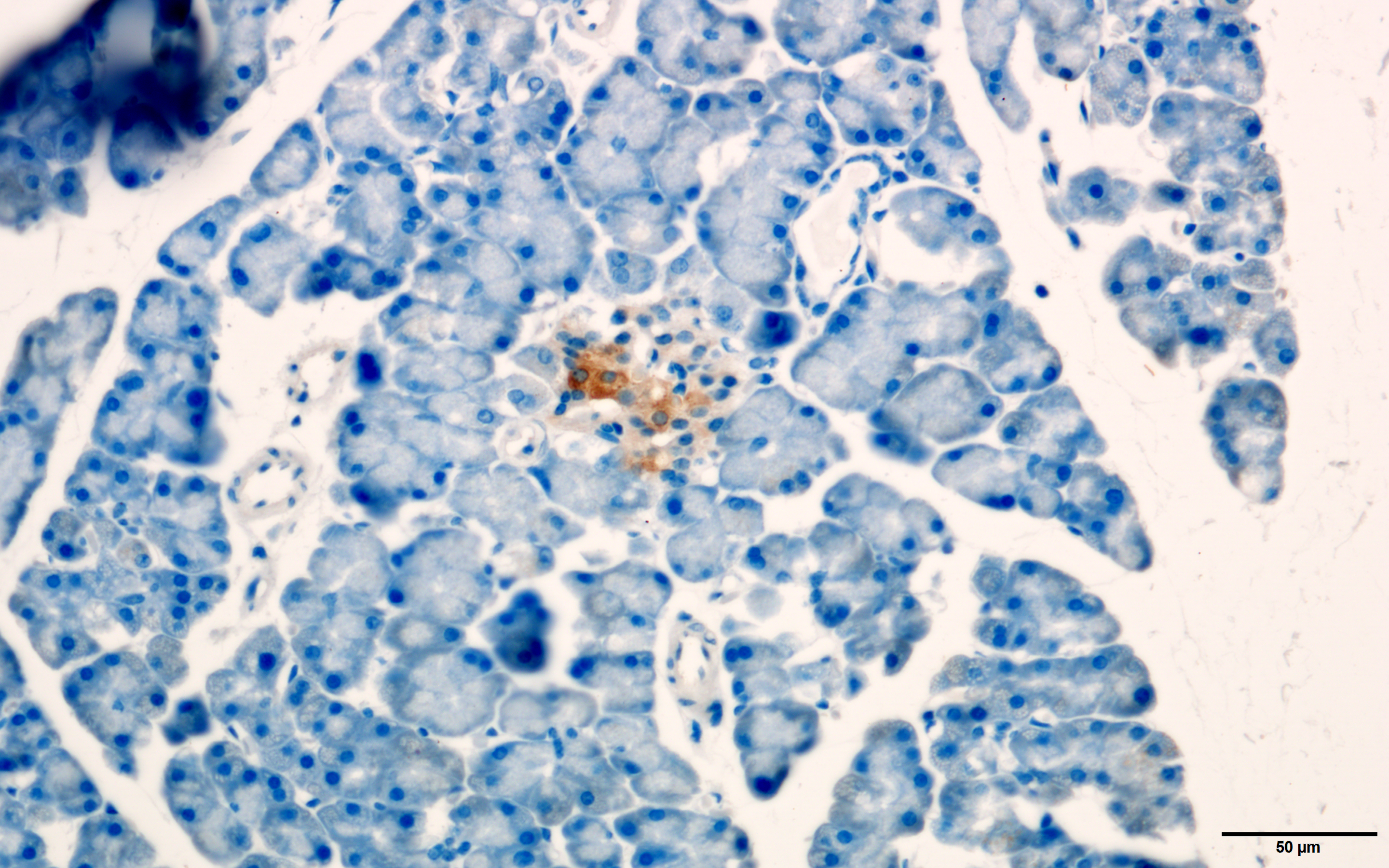

Supplement: Supplemental Information 11 [file peerj-11-15705-s011.zip › Fig.5 STZ400X.tif]

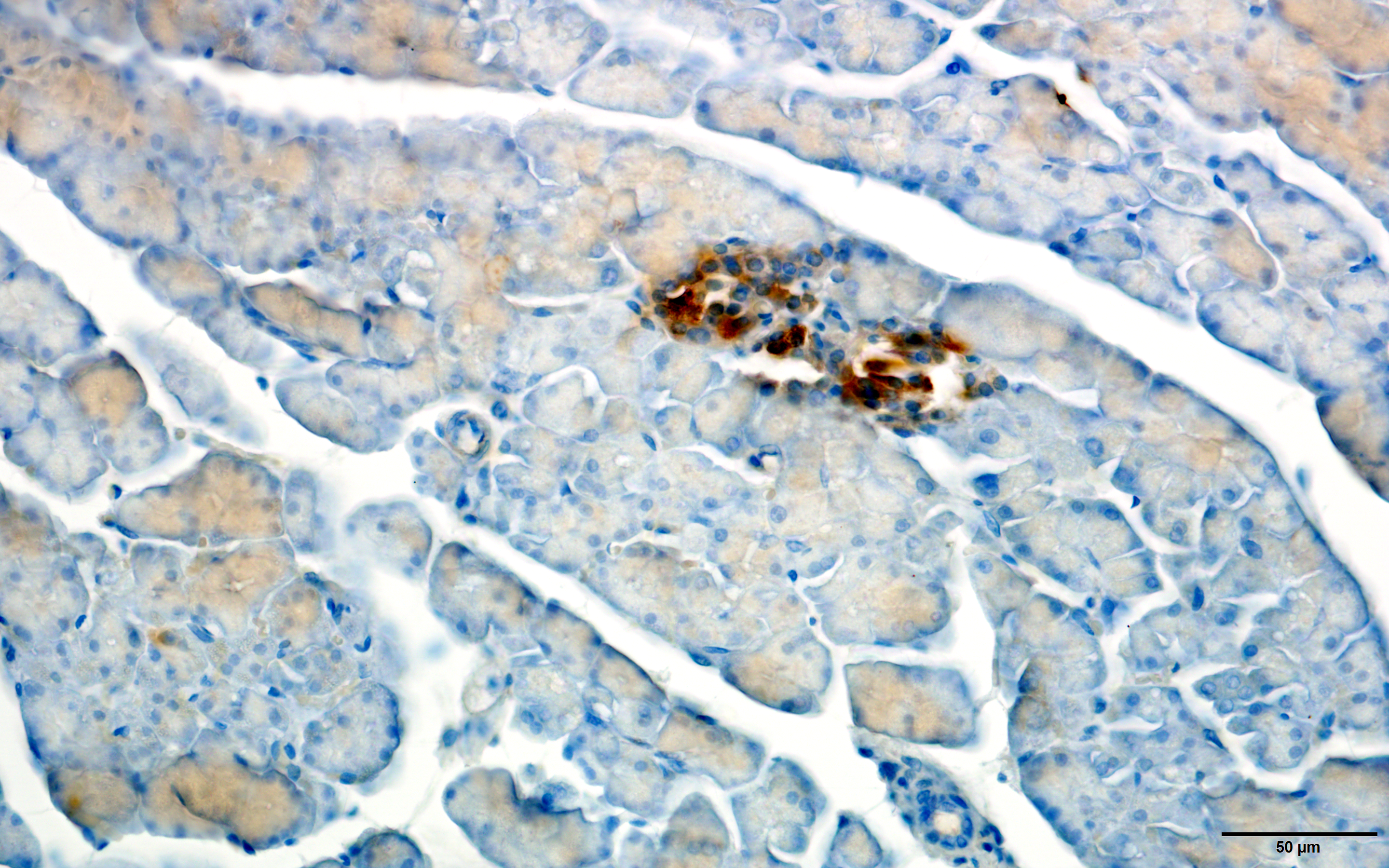

Supplement: Supplemental Information 11 [file peerj-11-15705-s011.zip › Fig.5 OA 400x.tif]

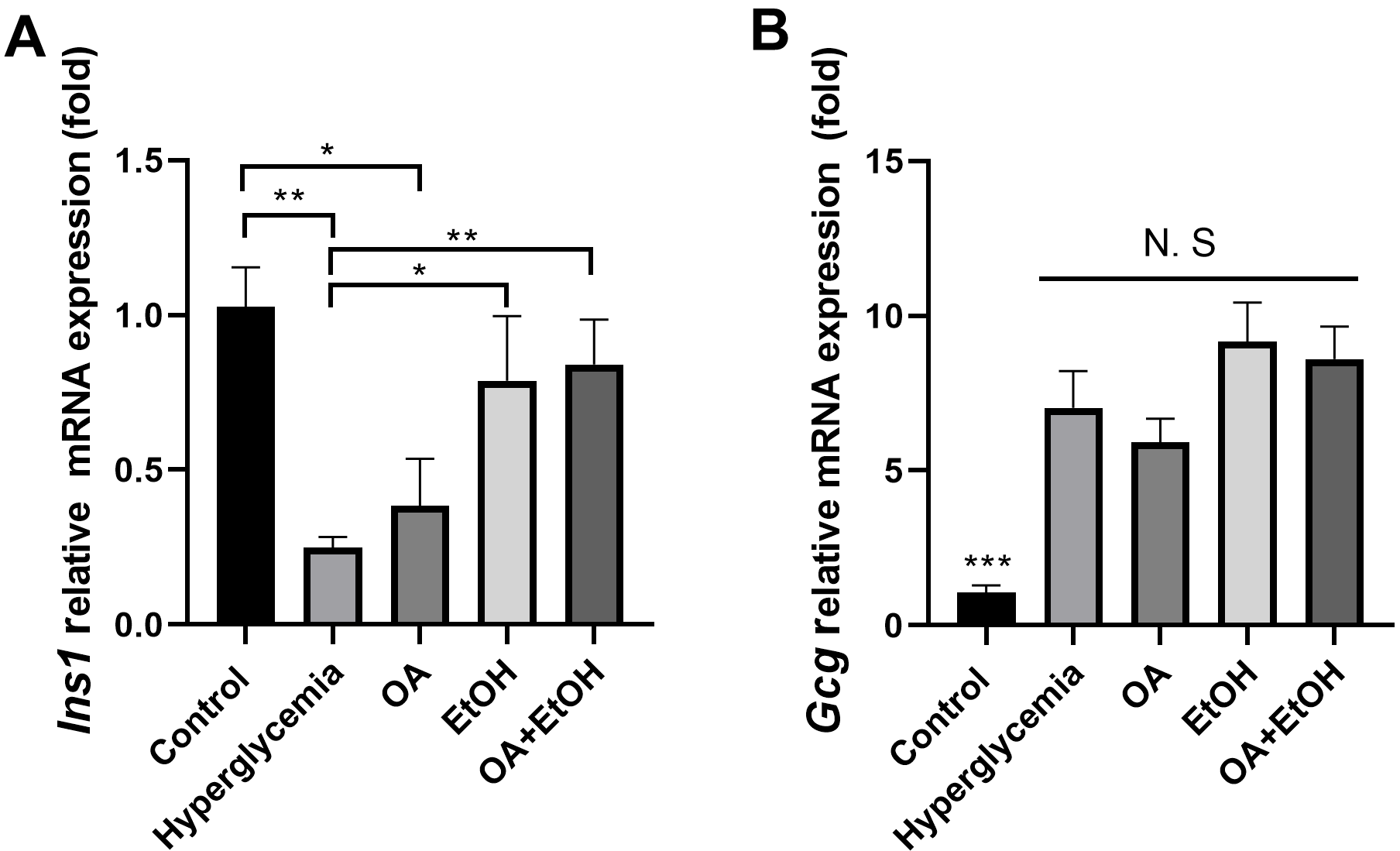

Supplement: Supplemental Information 12 [file peerj-11-15705-s012.zip › Fig.6 RNA expression/Fig.6.png]

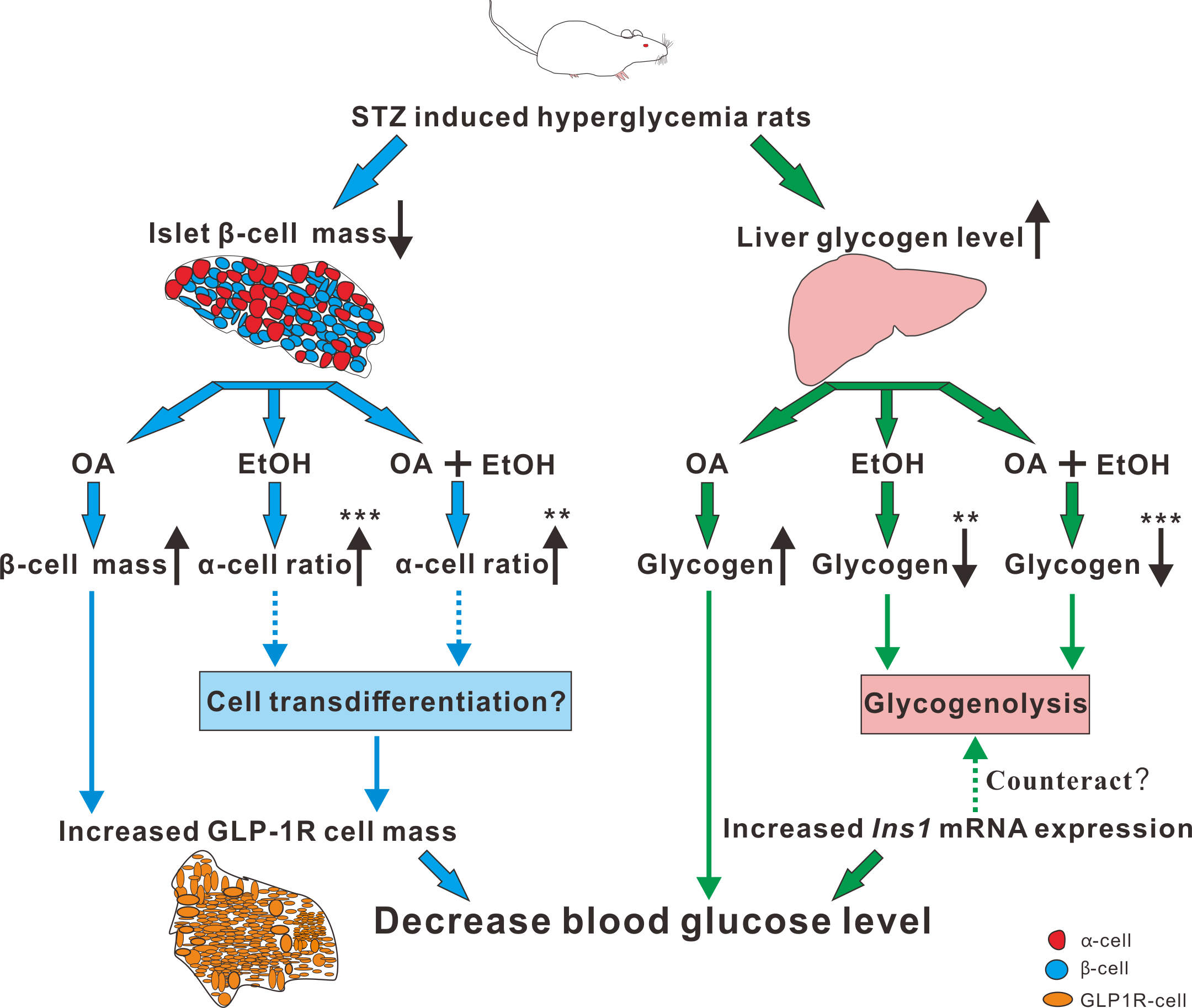

Supplement: Supplemental Information 13 [file peerj-11-15705-s013.zip › Fig.7 Summary figure/Figure7.png]
